# An Oriental Medicine, Hyungbangpaedok-San Attenuates Motor Paralysis in an Experimental Model of Multiple Sclerosis by Regulating the T Cell Response

**DOI:** 10.1371/journal.pone.0138592

**Published:** 2015-10-07

**Authors:** Jong Hee Choi, Min Jung Lee, Minhee Jang, Eun-Jeong Kim, Insop Shim, Hak-Jae Kim, Sanghyun Lee, Sang Won Lee, Young Ock Kim, Ik-Hyun Cho

**Affiliations:** 1 Department of Convergence Medical Science, College of Korean Medicine, Kyung Hee University, Seoul, 130–701, Republic of Korea; 2 Brain Korea 21 Plus Program, College of Korean Medicine, Kyung Hee University, Seoul, 130–701, Republic of Korea; 3 Acupuncture & Meridian Science Research Center, College of Korean Medicine, Kyung Hee University, Seoul, 130–701, Republic of Korea; 4 Department of Clinical Pharmacology, College of Medicine, Soonchunhyang University, Cheonan, 336–745, Republic of Korea; 5 Department of Integrative Plant Science, Chung-Ang University, Anseong, 456–756, Republic of Korea; 6 Department of Medicinal Crop Research Institute, National Institute of Horticultural & Herbal Science, Rural Development Administration, Eumseong, 369–873, Republic of Korea; 7 Institute of Koreran Medicine, College of Korean Medicine, Kyung Hee University, Seoul, 130–701, Republic of Korea; Research Inst. of Environmental Med., Nagoya Univ., JAPAN

## Abstract

The preventive and therapeutic mechanisms in multiple sclerosis are not clearly understood. We investigated whether Hyungbangpaedok-san (HBPDS), a traditional herbal medicine, has a beneficial effect in experimental autoimmune encephalomyelitis (EAE) mice immunized with myelin oligodendrocyte glycoprotein peptide (MOG_35-55_). Onset-treatment with 4 types of HBPDS (extracted using distilled water and 30%/70%/100% ethanol as the solvent) alleviated neurological signs, and HBPDS extracted within 30% ethanol (henceforth called HBPDS) was more effective. Onset-treatment with HBPDS reduced demyelination and the recruitment/infiltration and activation of microglia/macrophages in the spinal cord of EAE mice, which corresponded to the reduced mRNA expression of pro-inflammatory cytokines (TNF-α, IL–6, and IL–1β), iNOS, and chemokines (MCP–1, MIP–1α, and RANTES) in the spinal cord. Onset-treatment with HBPDS inhibited changes in the components of the blood-brain barrier such as astrocytes, adhesion molecules (ICAM–1 and VCAM–1), and junctional molecules (claudin–3, claudin–5, and zona occludens–1) in the spinal cord of EAE mice. Onset-treatment with HBPDS reduced the elevated population of CD4^+^, CD4^+^/IFN-γ^+^, and CD4^+^/IL–17^+^ T cells in the spinal cord of EAE mice but it further increased the elevated population of CD4^+^/CD25^+^/Foxp3^+^ and CD4^+^/Foxp3^+^/Helios^+^ T cells. Pre-, onset-, post-, but not peak-treatment, with HBPDS had a beneficial effect on behavioral impairment in EAE mice. Taken together, HBPDS could alleviate the development/progression of EAE by regulating the recruitment/infiltration and activation of microglia and peripheral immune cells (macrophages, Th1, Th17, and Treg cells) in the spinal cord. These findings could help to develop protective strategies using HBPDS in the treatment of autoimmune disorders including multiple sclerosis.

## Introduction

Multiple sclerosis (MS) is a T cell-mediated autoimmune disease of the central nervous system (CNS). T cells are activated in the peripheral immune system, recruited and infiltrate to the CNS, and are reactivated. These events cause demyelination and axonal loss in the CNS, eventually leading to paralysis and neurological disabilities [1]. In the current concept of pathogenesis of MS, CD4^+^ T cells can differentiate into various types of T helper (Th) cells, when activated by antigens, including the Th1, Th2, Th17 cells, and T regulatory (Treg) cells [1,2]. The Th cells that infiltrate into the CNS via a disrupted blood brain barrier encounter resident antigen-presenting cells in the CNS, secrete inflammatory mediators, and induce microglial activation. As a result, these cells produce factors that attract more immune cells into the CNS and sustain the inflammatory cascade [1,2,3]. However, the etiology of MS remains uncertain.

The current MS therapeutic drugs aim to prevent relapses and slow the progression of the disease. Several MS drugs have been approved as disease-modifying therapies that include interferon beta (IFN-β), glatiramer acetate, natalizumab, fingolimod, alemtuzumab, teriflunomide, and dimethyl fumarate. However, long-term use of MS drugs can cause various side effects. In addition, because the price of medicine is so expensive, MS patients suffer the economic problems [4,5]. Therefore, the use of natural products for the treatment of MS may be more effective and they have fewer side effects. Oriental herbal medicines have been reported to improve clinical symptoms, neurological signs, and immune function and reduce the frequency of recurrence in MS patients [6]. For example, Zuo-Gui and You-Gui pills, two traditional Chinese herbal formulas have been shown to reduce the clinical severity of MS [7,8,9] and suppress ongoing EAE [10]. These studies suggest that traditional Oriental herbal formulas may serve as new neuroprotective strategies for MS.

Hyungbangpaedok-san (HBPDS) is an herbal prescription that is extensively used in traditional Oriental medicine, which is composed of 10 kinds of herb; *Ostericum koreanum*, *Aralia continentalis*, *Bupleurum falcatum*, *Angelica decursiva*, *Schizonepeta tenuifolia*, *Saposhikovia divaricata*, *Poria cocos*, *Rehmannia glutinosa*, *Lycium barbarum*, and *Plantago asiatica*. HBPDS has traditionally been used for patients with fever and chills that are not sweating, pain, and stiffness in the head and neck, generalized body aches and pain, and redness and swelling of the eyes [11]. HBPDS has anti-convulsive activity, sedative activity, and anti-aging effect through the weight, hematologic, and biochemical changes in Wister rat model [12,13]. HBPDS decreases tumor necrosis factor-α (TNF-α) and signal transducer and activator of transcription 4 expression and increases proliferation of CD4^+^ T cells, which are associated with the anti-inflammatory activity and immunomodulatory effects [13,14]. These recent reports increase the possibility that HBPDS may be an effective treatment for patients with autoimmune diseases such as MS. To explore this possibility, we investigated whether HBPDS has an ability to improve neurological impairment and reduce spinal demyelination and inflammation in the myelin oligodendrocyte glycoprotein peptide (MOG_35-55_)-induced experimental autoimmune encephalomyelitis (EAE) mice model, an animal model of MS. We confirmed that HBPDS has a beneficial effect on neurological impairment, which correlated with reduced demyelination, diminished BBB disruption, and inhibited infiltration and recruitment of immune cells into the spinal cord. Our findings indicate that HBPDS could be applied as a neuroprotective strategy in patients with MS through further investigation.

## Materials and Materials

### 2.1. Animals and Ethics statement

The 8 to 9 weeks old C57BL/6 female mice (weight, 18–20 g) were purchased from the Narabiotec Co., Ltd. (Seoul, Korea). Animals were housed 6 per cage, allowed spontaneous take in food and water. Animals were kept under a 12-hour light/dark cycle (light on 07:00–19:00) at room temperature (23 ± 2°C) and humidity (55 ± 10%). All experimental procedures were reviewed and specifically approved by the Institutional Animal Care and Use Committee (IACUC) or Ethics Committee of Animal Experiments of Kyung Hee University, Republic of Korea (Permit Number: KHUASP/SE-12-029). In this process, proper randomization of laboratory animals and handling of data were performed in a blinded manner in accordance with recent recommendations from a NIH Workshop on preclinical models of neurological diseases [15].

### 2.2. Animal sacrifice

All efforts were made to prevent undue stress or pain to the mice. Mice with signs of imminent death were euthanized to avoid suffering. Mice were deeply anesthetized with an intraperitoneal overdose of sodium pentobarbital (50 mg/kg, body weight) and spinal cords were sampled.

### 2.3. Preparation of HBPDS extracts

Ten dried medicinal herbs (*Ostericum koreanum*, *Aralia continentalis*, *Bupleurum falcatum*, *Angelica decursiva*, *Schizonepeta tenuifolia*, *Saposhikovia divaricata*, *Poria cocos*, *Rehmannia glutinosa*, *Lycium barbarum*, and *Plantago asiatica*), components of HBPDS, were purchased from Omniherb (Daegu, Korea). Each dried herb was mixed at an equal weight (20 g each/200 g in total) and cut into small pieces. The mixture was incubated in 2.0 L distilled water using a reflux extraction system for 90 minutes and was boiled for 90 minutes. The aqueous extract was filtered through Whatman No. 4 filter paper having a pore size of 20–25μm and it was concentrated by vacuum evaporation using a EYELA N-1200A, (EYELA, Rikakikai Co. Ltd., Tokyo, Japan) at 60°C. The viscous extract was lyophilized and stored at -80°C until use. The solvents for extract were prepared with distilled water (DW), 30% ethanol (ethyl alcohol, EtOH) solution (v/v), 70% ethanol (v/v), and 100% ethanol. The final yields were 18.3%, 17.3%, 16.9%, and 5.2% in DW, 30% ethanol, 70% ethanol, and 100% ethanol, respectively. Total daily dose of HBPDS into animals was determined after considering body weight of animals, metabolic rate in animals, final extract yield, and traditional dose in humans.

### 2.4. Identification of HBPDS extract by qualitative HPLC analysis

HBPDS (extracted with 30% ethanol) was identified using high-performance liquid chromatography. A 1 g of HBPDS extract was dissolved in 200 ml of 70% methanol and filtered through a 0.45 μm polyvinylidene difluoride filter. The standard materials used for the qualitative analysis of HBPDS were oxypeucedanin (of *ostericum koreana*) and pachymic acid (of *poria cocos*). The standard stock solutions were prepared by dissolving 5 mg samples, each in 50 ml methanol. The samples were separated on a phenomenex luna C18 reverse phase C18 (250 x 4.6 mm, 5 μm) (Phenomenex, Torrance, CA, USA) at 25°C. Detection wave length was at UV 203 nm for saikosaponin A, 254 nm for oxypeucedanin and prim-O-glucosylcimifugin, 210 nm for pulegone, and 242 nm for pachymic acid. The mobile phase consisted of (A) acetonitrile/water (40/60, v/v) and (B) acetonitrile/water (90/10, v/v) at a flow-rate of 1.0 ml/minute. The solvent gradient elution program was as follows: A: B, 0 minute (100: 0), 20 minutes (75: 25), 35 minutes (25: 75), and finally 36 minutes (0: 100).

### 2.5. Experimental group, EAE induction, and clinical evaluation

**Fig 1:**
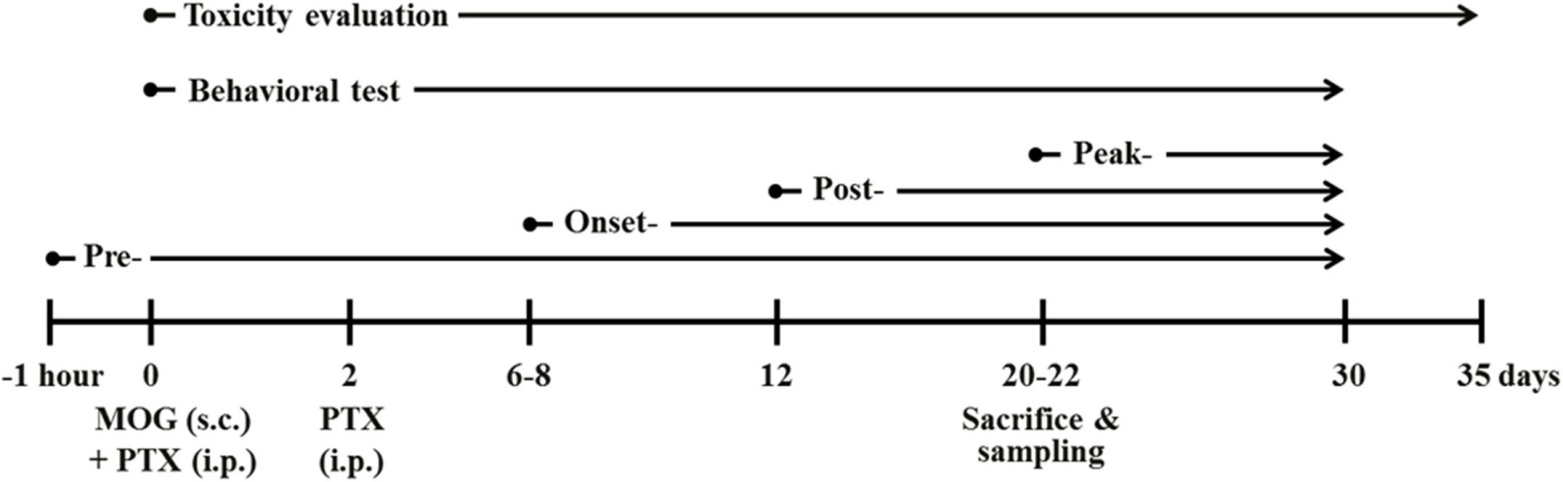
Schematic design of the experimental protocol. Mice were divided into the following four experimental groups: HBPDS-pretreated (daily from 1 hour before immunization), onset-treated (daily from about day 6–8 after immunization), post-treated (from day 12 after immunization), and peak-treated (from day 20–22 after immunization) groups. Mice in the EAE and EAE + HBPDS groups were subcutaneously immunized with MOG_35-55_ peptide. Behavioral test (recording of the clinical score) was evaluated daily at the same time for 30 days after immunization. For toxicity evaluation, mice were treated daily with 3 doses of HBPDS alone (1x, 2x, and 4x HBPDS) during the 5 weeks.

Basically, the experimental group was subdivided into the following groups (n = 8–10 per group): normal control group [vehicle treatment, s.c. + saline, p.o.], EAE [200 μg of MOG_35-55_, s.c. + saline, p.o.], EAE + HBPDS group [200 μg of MOG_35-55_, s.c. + 10 or 20 mg/kg of DW-extracted HBPDS, 15 or 30 mg/kg of 30% ethanol-extracted HBPDS, 15 or 30 mg/kg of 70% ethanol-extracted HBPDS, or 5 or 10 mg/kg of 100% ethanol-extracted HBPDS, p.o.], and HBPDS alone group [vehicle treatment, s.c. + HBPDS, p.o.]. Mice were immunized with subcutaneously with 100 μl of an emulsion containing 200 μg of MOG_35-55_ (Sigma-Aldrich, St. Louis, MO, USA) in phosphate buffered saline (PBS), equivalent volumes of incomplete Freund’s adjuvant (Difco, Detroit, MI, USA), and 550 μg of *mycobacterium tuberculosis* H37Ra (Difco) into the hind flanks. Mice received intraperitoneally, injections of 250 ng of pertussis toxin (PTX; List Biologic, Campbell, CA, USA) on day 0 of immunization and day 2 after immunization (Fig 1). Mice in the normal group were treated with saline alone instead of MOG_35-55_ peptide or PTX. Clinical signs of EAE were evaluated daily and scored using the clinical scoring scale as previously described [16,17]: grade 0, absence of symptoms; grade 1, partial loss of tail tonus; grade 2, paralysis of tail; grade 3, paraparesis; grade 4, paraplegia; grade 5, tetraparesis; grade 6, tetraplegia; grade 7, death.

### 2.6. Histopathological evaluation

On the peak days (20–22 days) of neurological impairment after immunization, mice were deeply anesthetized, perfused intracardially with 0.9% saline followed by 4% paraformaldehyde (PFA) in 0.2 M phosphate buffer (PB, pH 7.4). Lumbar spinal cords were removed, fixed using 4% PFA for a day, incubated overnight in 30% sucrose solution, and then cut into 10-μm thick by previously described [16,17]. The sections were stained with luxol fast blue (LFB) and hematoxylin and eosin (H&E) to evaluate demyelination and immune cell infiltration, respectively. The sections were dehydrated and coverslipped as previously described [16,17].

The level of demyelination after LFB staining was evaluated as previously described [18]: 0, no demyelination; 1, little demyelination, only around infiltrates and involving less than 25% of the white matter; 2, demyelination involving less than 50% of the white matter; 3, diffuse and widespread demyelination involving more than 50% of the white matter. The level of recruitment/infiltration of immune cells after H&E staining was scored according to the following criteria [18]: 0, no lesion; 1, cellular recruitment/infiltration only in the meninges; 2, very discrete and superficial infiltrates in parenchyma; 3, moderate infiltrate (less than 25%) in the white matter; 4, severe infiltrates (less than 50%) in the white matter; 5, more severe infiltrates (more than 50%) in the white matter.

### 2.7. Immunohistochemistry

Immunohistochemical analysis was accomplished as previously described [16,17,19] using rabbit anti-ionized calcium binding adaptor molecule–1 (Iba–1) (1:2,000; WAKO, Osaka, Japan) or rabbit anti-glial fibrillary acidic protein (GFAP) (1:2,000; DACO, USA). The sections were incubated with incubated with biotinylated rabbit IgG antibody (1:200; Vector Laboratories, USA) for 1 hour at room temperature, incubated with avidin-biotinylated horseradish peroxidase-complex (1:200; Vector Laboratories) for 1 hour at room temperature and, visualized with 3,3‘-diamino-benzidine. Sections were rinsed and dehydrated and cover slipped.

### 2.8. Reverse transcription-polymerase chain reaction (RT-PCR)

After the peak stage (20–22 days) of neurological impairment after immunization, spinal cords were harvested and total RNA was extracted from spinal cord using TRIsure reagent according to the manufacturer’s instructions (Bioline, UK). cDNA was synthesized by incubating 1 μg of total RNA for 1 hour at 37°C in a reaction mixture containing 0.5 μg of Oligo dT, 0.5 mM dNTP mix, 5x first-strand buffer, RNase out, 5 mM dithiothreitol (DTT), and M-MLV reverse transcriptase. RT-PCR analysis was performed according to the manufacturer’s instructions (RT-PCR kit; Roche, Germany). Sequences of PCR primers were listed in S1 Table. For PCR amplification, specific oligonucleotide primer pairs were incubated with 1 μl of cDNA and 0.6 U of Econo TaqDNA polymerase in 6 μl of PCR Master Mix (Lucigen, WI, USA). Saturation curves for PCR were obtained from various experimental conditions (RNA concentrations, annealing temperatures, and PCR cycle numbers). We determined the optimal amplification conditions (annealing temperature and PCR cycle number) of primers for the PCR. The 5 to 7 μl of the resulting mix were then electrophoresed on a 2% agarose gel and visualized by ethidium bromide staining on a transilluminator. Expression levels of each gene were normalized to that of glyceraldehyde 3-phosphate dehydrogenase (GAPDH).

### 2.9. Real-time PCR

To investigate the mRNA level by real-time PCR analysis, cDNA was synthesized according to same method with RT-PCR analysis as outlined above. Real-time PCR was accomplished using SYBR Green PCR Master Mix (Applied Biosystems, Franklin Lakes, NJ, USA) as previously described [16,19]. In brief, each ingredient for PCR amplification was involved as 1 μl of 5 pM primer, 4 μl of cDNA and 5 μl of SYBR Green in total volume of 10 μl. Numerical value of mRNA levels was normalized to that of GAPDH. Fold-induction was calculated using the 2^−ΔΔCT^ method. PCR amplification was performed at least three times. Sequences of oligonucleotide primers were listed in S1 Table.

### 2.10. Flow cytometry

For flow cytometry analysis, six mice in each group at peak period (day 20–22) after immunization were anesthetized and lumbar spinal cord and lymph node were carefully dissected and dissociated as previously described [16,17]. Briefly, single-cell suspensions refined from whole tissue were prepared (centrifuged at 300 g for 5 minutes) and fixed with 2% PFA, cells were washed with washing buffer containing 2% FBS in PBS, incubated with mouse anti-rat CD32 (BD Bioscience) for 10 minutes to block the Fc receptor and washed twice with 2% FBS washing buffer. For cell surface staining of immune markers with fluorescently labeling, the cells were incubated with APC anti-mouse CD4 (RM4-5, BD Biosciences), PE anti-mouse CD8a (53–6.7, BD Biosciences), APC anti-mouse/human CD11b (M1/70; Biolegend), and PE anti-mouse CD45 (30-F11; BD Biosciences) for 30 minutes at 4°C. Non-stained cells were used as negative controls. For intracellular cell staining, cells were restimulated with PMA (phorbol 12-myristate-13-acetate, Sigma), ionomycin (Sigma), and Golgistop (protein transport inhibitor, BD Biosciences) for 5 hours. After this stimulation, cells were fluorescent stained with PerC3 anti-mouse CD4 (RM4-5, BD Biosciences), FITC anti-mouse IFN-γ (BD Biosciences), PE anti-mouse IL-17A (TC11-18H10, BD Biosciences), PE anti-mouse IL–4 (11B11; Biolegend), PE anti-mouse CD25 (PC61.5; eBiosciences), APC anti-mouse/rat forkhead box P3 (Foxp3) (FJK-16s; eBioscience), or PE anti-mouse Helios (22F8; BD Biosciences) for 30 minutes at 4°C. The stained cells were washed twice with 2% FBS washing buffer and used for flow cytometry. Data were collected on a FACSCalibur flow cytometer (BD Biosciences) and analyzed using CellQuest Pro software (BD Biosciences). CD4^+^ T cells were gated to analyze populations of Th1, Th2, Th17, and Treg cells. Three-color staining of one cell for simultaneous analysis and results of intracellular cytokines were indicated as the percentage within the CD4^+^ population. We acquired through set up of 10,000 cells events, and gating was set by side scatter.

### 2.11. Immunoblotting

The spinal cord tissues were dissected from the mice at peak period (day 20–22 after immunization). Tissues were homogenized, proteins were prepared and transferred, and immunoblot analysis was accomplished as previously described [16,17,19] using glyceraldehyde-3-phosphate dehydrogenase (GAPDH), mouse anti-glial fibrillary acidic protein (GFAP), rabbit anti-myelin basic protein (MBP) (1:1,000; Millipore), and rabbit anti-ionized calcium binding adaptor molecule 1(Iba–1) (1:500, WAKO) antibodies.

### 2.12. Statistical analysis

Statistical analysis was performed by using the SPSS 21.0 package (SPSS Inc, Chicago, USA) for Windows. All of the data were presented as mean ± SEM. Sum of neurological score, histological score, and immunological assays were compared using the one-way ANOVA with Tukey *post hoc* test for comparison of multiple groups. For comparison of sum of neurological score, between two different treatments, statistical analysis was done by two-tailed Student’s t-test. The P values of less than 0.05 were accepted as statistically significant.

## Results

### 3.1. Onset-treatment with HBPDS has beneficial effect for neurological signs in EAE mice

**Fig 2:**
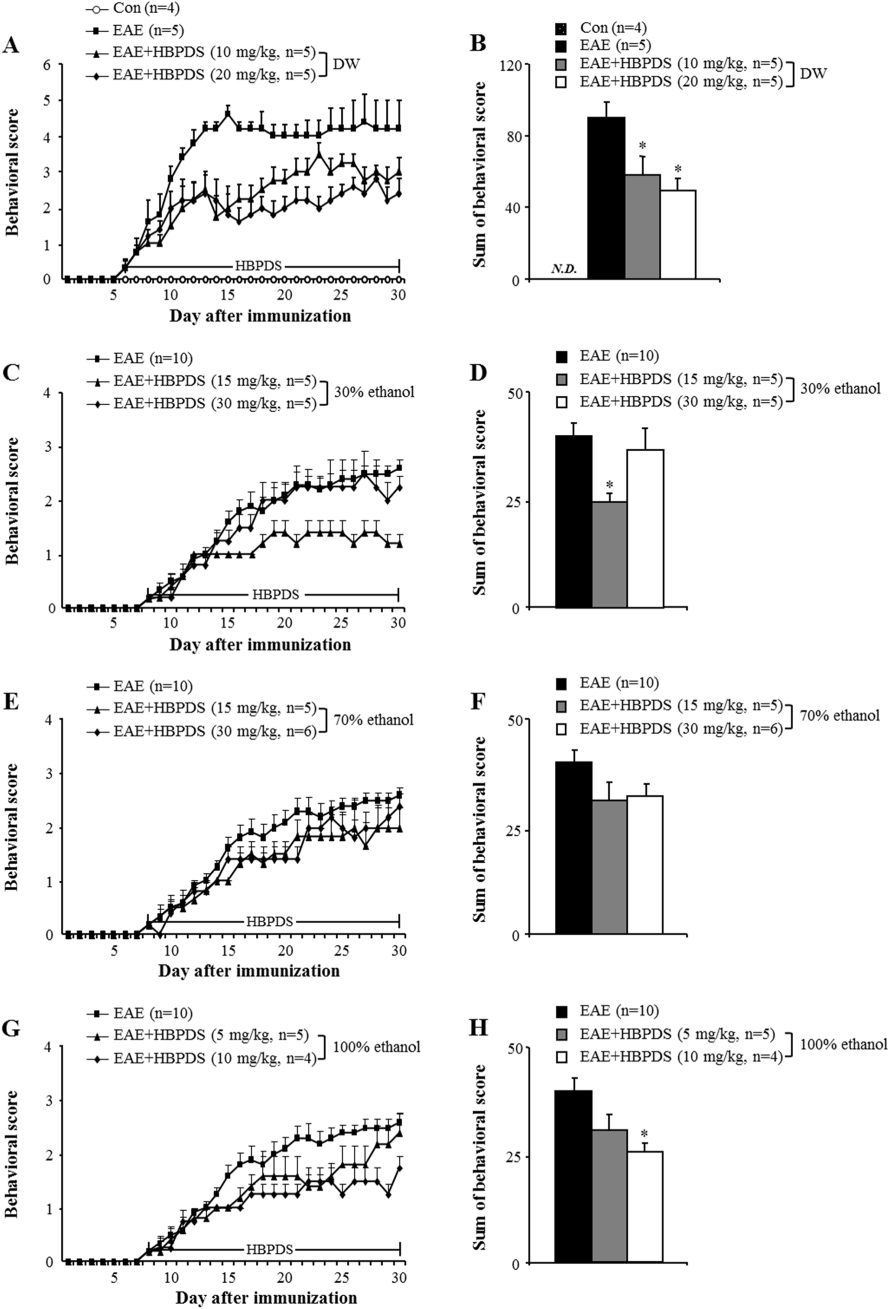
HBPDS alleviates neurological impairment in EAE mice. **(A-H)** Onset-treatment with various extract types of HBPDS has beneficial effects on neurological impairment in EAE mice. DW-extracted (10 and 20 mg/kg) (A and B), 30% ethanol-extracted (15 mg/kg) (C and D), 70% ethanol-extracted (30 mg/kg) (E and F), and 100% ethanol extracted HBPDS (10 mg/kg) (G and H) reduced the development and severity of neurological impairment. B, D, F, and H indicate the sum of daily scores after onset-treatment with HBPDS. Behavioral score value of mice in the EAE group shown in Figure C-G was commonly used. Data are expressed as mean behavioral score ± SEM. (ANOVA test; *p < 0.05 versus EAE group, ##p < 0.05 versus EAE group).

**Fig 3:**
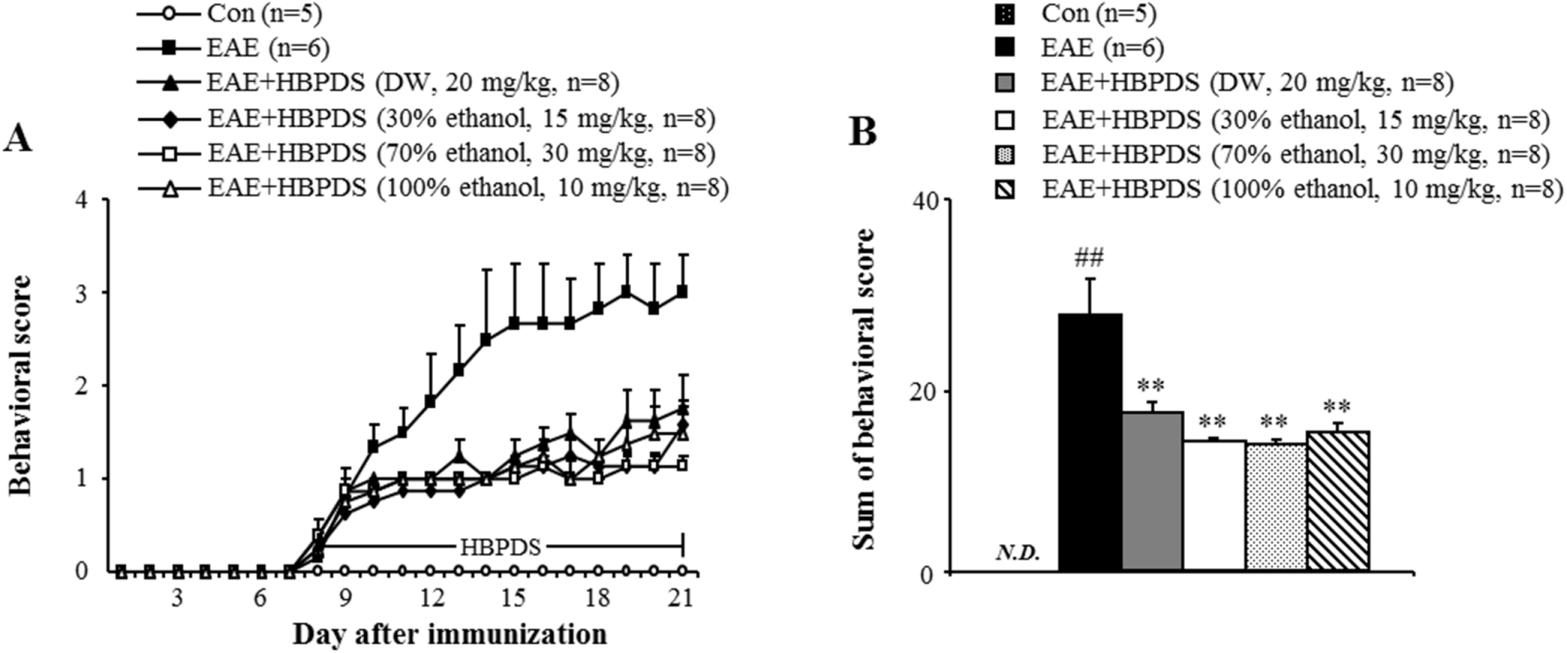
The 4 extract types of HBPDS have a similar effect on neurological impairment in EAE mice. **(A-D)** To investigate the most efficacious extract of HBPDS for treating neurological impairment in EAE mice, the 4 conditions selected according to Fig 2 were compared. Onset-treatment with DW- (20 mg/kg), 30% ethanol- (15 mg/kg), 70% ethanol (30 mg/kg), and 100% ethanol extracted (10 mg/kg) HBPDS had a similar effect on neurological impairment (A and B) in EAE mice; however, 30% and 100% ethanol-extracted HBPDS were slightly more effective. Sum of behavioral score (B) indicates the sum of score after onset-treatment with HBPDS. Data are expressed as mean scores ± SEM. (ANOVA test; ##p < 0.01 compared to normal control mice, **p < 0.01 versus EAE mice).

**Fig 4:**
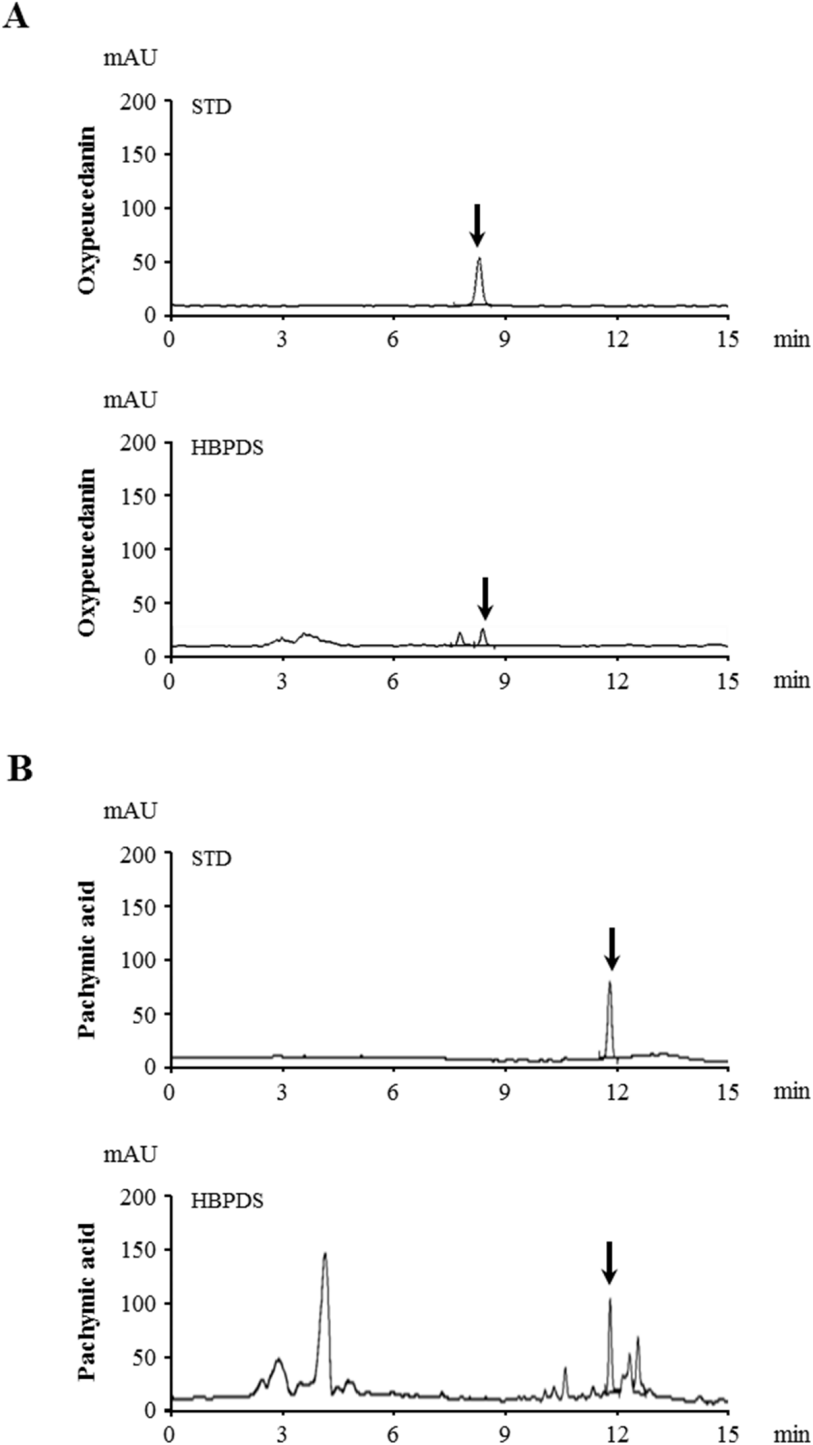
Quantitative HPLC analysis of the 30% ethanol extract of HBPDS. Peaks for oxypeucedanin (A) and pachymic acid (B) corresponded to standard at 8.393 minutes and 11.782 minutes, respectively.

First, we investigated the optimum dosage of HBPDS that produces the most significant effect on the neurological symptoms of EAE (Fig 2). In mice from the EAE group, the mean behavioral score gradually increased starting at day 6–8 after immunization, peaked at day 15–20 after immunization, and gradually declined thereafter, compared with that in the normal control group. However, onset-treatment (daily from day 6–8 after immunization) with 4 types of HBPDS [DW (Fig 2A and 2B), 30% (Fig 2C and 2D), 70% (Fig 2E and 2F), or 100% (Fig 2G and 2H) ethanol-extracted] and 2 doses per type of HBPDS reduced the severity of neurological impairment compared to that in EAE mice. Onset-treatment with 20 mg/kg of DW-, 15 mg/kg of 30% ethanol-, 30 mg/kg of 70% ethanol-, and 10 mg/kg of 100% ethanol-extracted HBPDS had more beneficial effects than the other doses in reducing the severity of neurological signs compared to that in EAE mice (*P* < 0.05). Subsequently, in Fig 3, we demonstrated more effective extract condition of HBPDS for reducing the severity of neurological signs in EAE mice. Although all 4 types of HBPDS had beneficial effects in EAE mice, 30% ethanol-extracted HBPDS (15 mg/kg) was the most effective in EAE mice. Based on these results, 30% ethanol-extracted HBPDS (15 mg/kg) was used in further study. Therefore, the qualitative determination of 30% ethanol-extracted HBPDS was performed by HPLC. The 30% ethanol-extracted HBPDS was standardized with oxypeucedanin (of ostericum koreana) and pachymic acid (of poria cocos) for quality consistency. Peaks for oxypeucedanin (Fig 4A) and pachymic acid (Fig 4B) corresponded to standard at 8.393 minutes and 11.782 minutes, respectively.

### 3.2. HBPDS has a wide neuroprotective time-window in EAE mice

**Fig 5:**
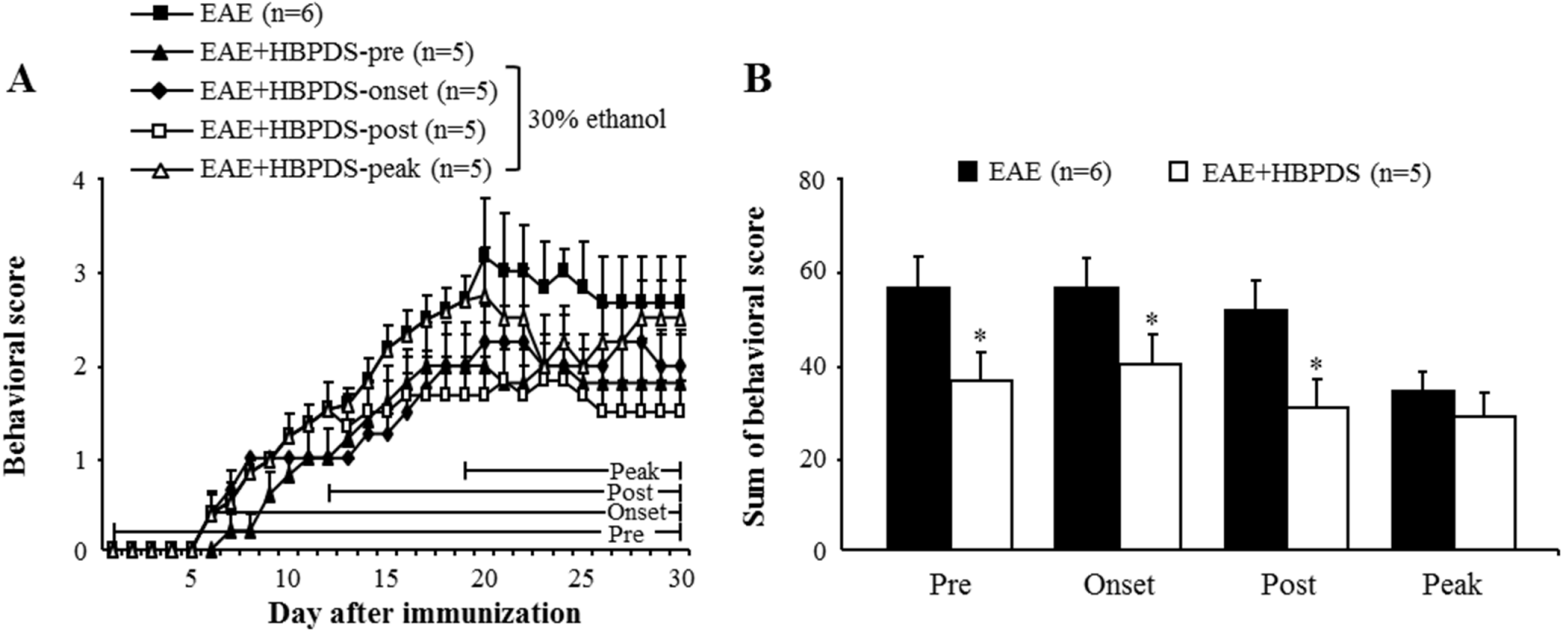
HBPDS has a wide neuroprotective time-window in EAE mice. **(A-B)** Pre-, onset-, and post-treatment but not peak-treatment with HBPDS (30% ethanol extracted) alleviated neurological impairment in EAE mice. Sum of behavioral score indicates the sum of scores of after pre-, onset-, post-, and peak-treatment with HBPDS (30% ethanol extracted) (B). Data are expressed as mean scores ± SEM. (Two-tailed Student’s t-test; *p < 0.05 compared to EAE mice).

To investigate neuroprotective time-window of HBPDS, 30% ethanol-extracted HBPDS was pretreated (daily from 1 hour before immunization), onset-treated (daily from day 6–8 after immunization), post-treated (daily from day 12 after immunization), and peak-treated (daily from the peak day of neurological score by immunization) in EAE mice. EAE mice appeared the neurological symptoms about day 6 and peak-point about day 21 after immunization. However, pre-, onset, and post-treatment with HBPDS significantly reduced the severity of neurological symptoms (Fig 5A) and decreased sum of daily average neurological score (Fig 5B) compared to the EAE mice. The maximum mean neurological score was 3.17 ± 0.6 in EAE mice and 2.0 ± 0.4, 2.25 ± 0.4, and 1.83 ± 0.4 in HBPDS pre-, onset, and post-treated mice, respectively. However, peak-treatment with HBPDS did not significantly reduce the average neurological score of EAE mice (Fig 5B). These results suggest that HBPDS has a wide neuroprotective time-window for neurological impairment in EAE mice.

### 3.3. HBPDS reduces demyelination and inflammation in the spinal cord of EAE mice

**Fig 6:**
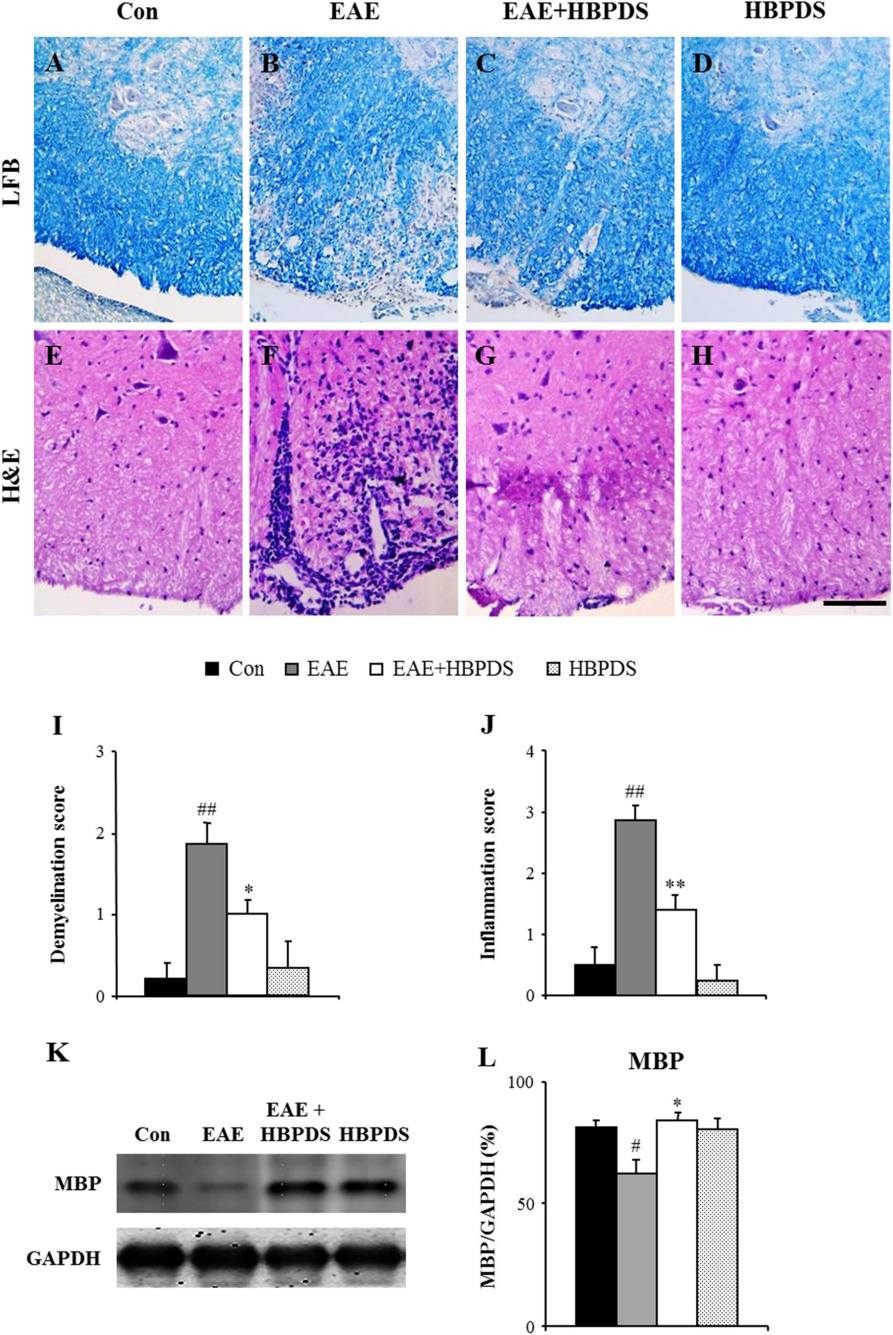
Onset-treatment with HBPDS reduces inflammation in the spinal cord of EAE mice. **(A-J)** Spinal cord sections obtained from mice in each group at day 20–22 after immunization were stained with luxol fast blue dye to evaluate the level of demyelination (A-D) and they were stained with eosin and hematoxylin to evaluate recruitment/infiltration of inflammatory cells (E-H), and quantified (I and J). Representative photographs show ventrolateral white matter of the spinal cord. Onset-treatment with HBPDS (30% ethanol extracted) decreased demyelination and recruitment/infiltration of inflammatory cells in the spinal cord of EAE mice compared with the spinal cord of EAE mice. **(K and L)** Spinal cord lysate was analyzed by immunoblotting (K) to evaluate the expression of MBP and the density was quantified (L). Data are expressed as the ratio of MBP to GAPDH for each sample. Scale bar = 100 μm. (ANOVA test; ##p < 0.01 compared to normal control mice, **p < 0.01 compared to EAE mice).

Since the onset point of neurological signs in MS/EAE is important for diagnosis and treatment [20] and onset-treatment with HBPDS markedly alleviated the severity of neurological impairment in EAE mice (Fig 5), we further studied the effect of onset-treatment with HBPDS in EAE mice. First, we investigated the relationship between the extent of neurological deficit and the level of demyelination in the spinal cord. On the peak-day (20–22 days) of neurological impairment, LFB staining showed prominent demyelination in the spinal cord from EAE mice (Fig 6B and 6I; demyelination score, 1.86 ± 0.26) compared to normal control mice, while the spinal demyelination was significantly decreased by onset-treatment with HBPDS (Fig 6C and 6I; demyelination score, 1.0 ± 0.18). In accordance with this morphological result, the protein expression of MBP was markedly decreased in the spinal cord of EAE mice (Fig 6K and 6L) compared to normal control mice, whereas these decreased expressions were significantly recovered by onset-treatment with HBPDS. Vehicle or HBPDS alone-treated mice did not induce demyelination (Fig 6A and 6D). To consecutively identify the level of infiltration of inflammatory cells, the spinal cords were stained with H&E on the peak-day (day 20–22 after immunization) of neurological impairment (Fig 6E–6H) and the level of infiltration of inflammatory cells was quantified (Fig 6J). Infiltration of inflammatory cells was increased in the white matter of the spinal cord of EAE mice (Fig 6F and 6J); however, the increased infiltration was decreased by onset-treatment with HBPDS (Fig 6G and 6J). These results suggest that HBPDS inhibits inflammation as well as demyelination in the spinal cord of EAE mice.

### 3.4. HBPDS reduces the mRNA expression of inflammatory mediators in the spinal cord of EAE mice

**Fig 7:**
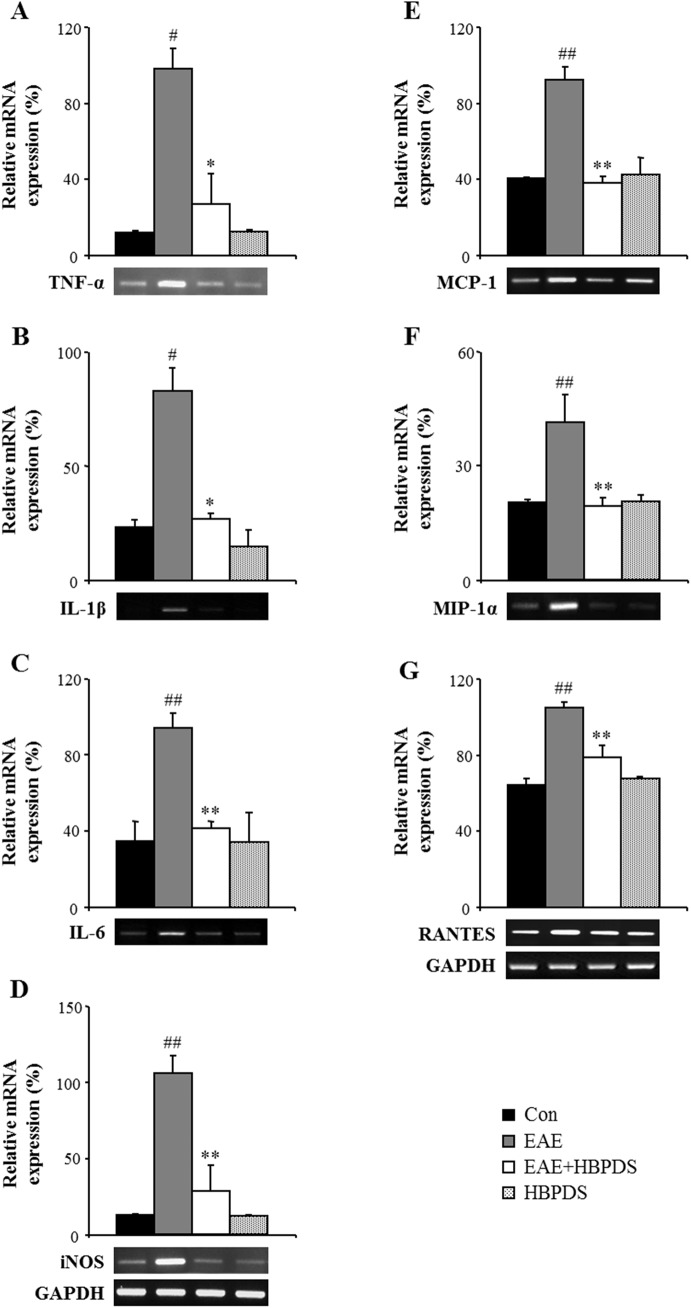
Onset-treatment with HBPDS decreases the mRNA expression of inflammatory mediators in the spinal cord from EAE mice. **(A-G)** Using total RNA isolated from the lumbar spinal cord of mice in each group at day 20–22 after immunization, the level of mRNA expression of inflammatory cytokines (A-C), iNOS (D), and chemokines (E-G) was measured by RT-PCR (bands in the bottom) and quantified (graphs). The mRNA expression levels of each gene were normalized to that of GAPDH. The mRNA expression levels of TNF-a (A), IL-1b (B), IL–6 (C), iNOS (D), MCP–1 (E), MIP–1α (F), and RANTES (G) were significantly decreased in the spinal cord of EAE mice by onset-treatment with HBPDS (30% ethanol extracted). Data are expressed as mean ± SEM (ANOVA test; ##p < 0.01 compared to normal control mice, **p < 0.01 compared to EAE mice).

In MS/EAE, neuronal degeneration is caused by inflammatory mediators released from infiltrated immune cells such as microglia, macrophages, and T cells [21]. Therefore, we examined the effect of HBPDS on the expression of representative inflammatory mediators in the spinal cord of EAE mice by RT-PCR analysis. In the spinal cord of EAE mice, the mRNA expression levels of pro-inflammatory cytokines (TNF-α, IL–1β, and IL–6) and iNOS were significantly increased compared to those in the normal control group; however, the increased mRNA expressions were significantly inhibited by onset-treatment with HBPDS (Fig 7A–7D). The mRNA expression of leukocyte recruitment-associated chemokines (MCP–1, MIP–1α, and RANTES) was also increased in the spinal cord of EAE mice; however, the increased expression was down-regulated by onset-treatment with HBPDS compared to that in the EAE group (Fig 7E–7G). These results suggest that HBPDS plays an important role in alleviating inflammation by reducing the inflammatory mediators released in the spinal cord of EAE mice.

### 3.5. HBPDS inhibits microglial activation in the spinal cord of EAE mice

**Fig 8:**
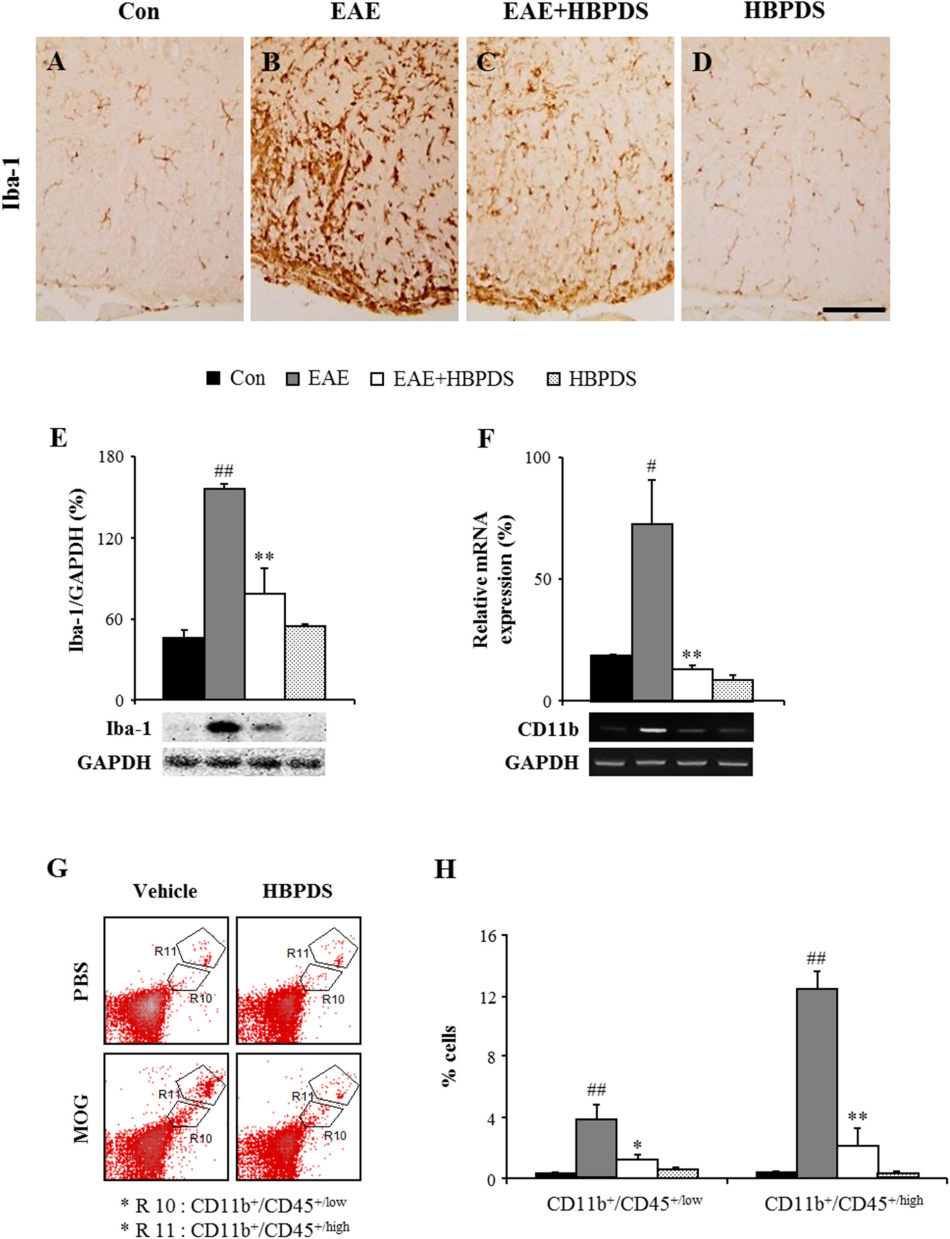
Onset-treatment with HBPDS reduces the infiltration and activation of microglia/macrophages in the spinal cord of EAE mice. **(A-F)** To investigate the level of infiltration and activation of microglia/macrophages on day 20–22 after immunization, lumbar spinal cord sections were analyzed by immunohistochemistry (A-D) and Western blots (E) using anti-Iba–1 antibody (A-E) and total RNA isolated from the lumbar spinal cord was analyzed by RT-PCR with primer for CD11b (F). Bands by Western blots and RT-PCR analysis were quantified as the ratio of Iba–1 (E) or CD11b (F) to GAPDH for each sample. The infiltration and activation of Iba–1 positive microglia/macrophages, the protein expression of Iba–1, and the mRNA expression of CD11b were significantly decreased by onset-treatment with HBPDS (30% ethanol extracted) in the spinal cord of EAE mice. **(G and H)** To further investigate the degree of recruitment/infiltration of microglia and macrophages, the lumbar spinal cord of mice in each group on day 20–22 after immunization was analyzed by flow cytometry. These cells were categorized into CD11b^+^/CD45^+/low^ cell (R10; microglia) and CD11b^+^/CD45^+/high^ cell (R11; macrophages) populations based on the CD45 expression levels (G) and the graph shows the percentage of each cell population (H). Onset-treatment with HBPDS (30% ethanol extracted) decreased the population of microglia and macrophages in the spinal cord with EAE mice. Data are presented as mean ± SEM. Scale bar = 100 μm. (ANOVA test; #p < 0.05, ##p < 0.01 compared to normal control mice, *p < 0.05 and **p < 0.01 compared to EAE mice).

Microglia are activated in inflammatory areas or demyelinating lesions in the brain and spinal cord of MS patients. Activated microglia and their products mediate progression of the disease [22]. Therefore, we investigated whether onset-treatment with HBPDS can regulate microglial activation in EAE mice. In the spinal cord of normal control or HBPDS alone-treated mice, Iba–1 (a specific marker for microglia/macrophage cells) positive cells showed the resting form of microglia with a small cell body and thin processes [23] (Fig 8A and 8D). However, in the spinal cord of EAE mice, Iba-1-positive cells were extensively activated and infiltrated. The activated microglia had an enlarged cell body with short and thick processes [23] (Fig 8B). Interestingly, the level of activated microglia was significantly reduced by onset-treatment with HBPDS compared to that in EAE mice (Fig 8C). The results corresponded to the change in the protein expression of Iba–1 by immunoblot analysis and the mRNA expression of CD11b by RT-PCR analysis in the spinal cord (Fig 8E and 8F). Our findings suggest that onset-treatment with HBPDS suppressed microglial activation and infiltration in the spinal cord of EAE mice.

### 3.6. HBPDS inhibits the infiltration of resident and peripheral immune cells in the spinal cord of EAE mice

Peripheral macrophages migrate and infiltrate into the inflammatory site of the CNS, and they contribute to pro- and anti-inflammatory reactions that interact with resident microglia in the lesion [24]. Therefore, we evaluated whether HBPDS is capable of suppressing the migration and infiltration of macrophages into the spinal cord of EAE mice. After the peak stage of neurological symptoms, the spinal cord was isolated from mice in each group and the population of resident microglia and peripheral macrophages was evaluated by the flow cytometry assay (Fig 8G and 8H). The population of CD11b^+^/CD45^+/high^ cells, representing the infiltrated macrophages, was markedly increased in EAE mice (12.39 ± 1.21%) compared to normal control mice (0.35 ± 1.21%). The enhanced population of macrophages was dramatically suppressed by HBPDS treatment (2.12 ± 1.27%) compared to that in EAE mice (Fig 8G and 8H). Moreover, the population of CD11b^+^/CD45^+/low^ cells, representing the microglia, was significantly increased in the spinal cord of EAE mice (3.95 ± 1.0%) compared to normal control mice (0.3 ± 0.08%); however, the increased population was significantly decreased in HBPDS-treated EAE mice (1.24 ± 0.35%), compared to EAE mice (Fig 8G and 8H). The results showed that HBPDS is able to reduce the severity of EAE through inhibiting the migration and infiltration of resident microglia and peripheral macrophages in the spinal cord of EAE mice.

### 3.7. HBPDS regulates the differentiation and recruit/infiltration of Th1, Th17, and Treg cells in EAE mice

**Fig 9:**
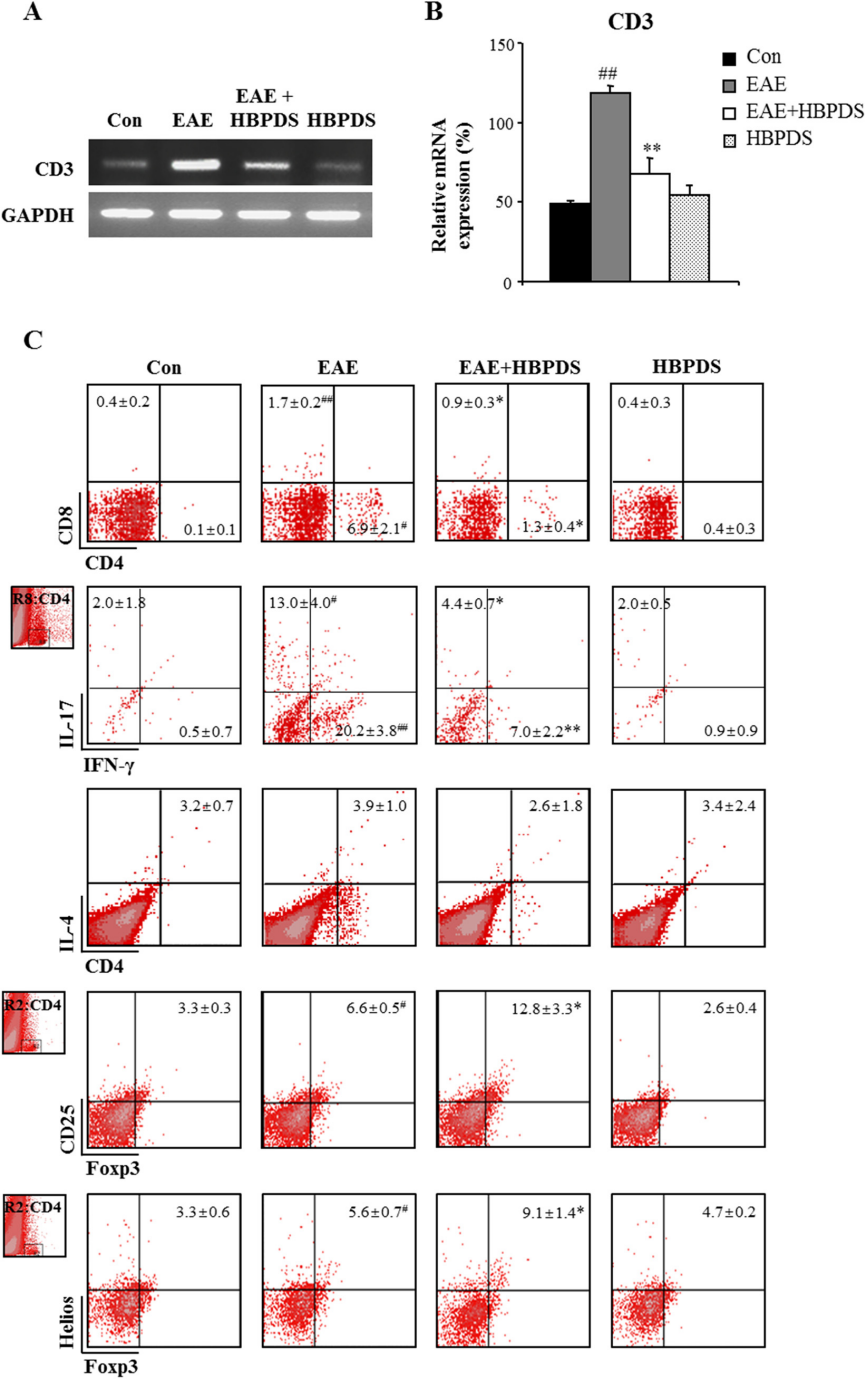
Onset-treatment with HBPDS regulates the recruitment/infiltration of CD4 T, Th1, Th17, and Treg cells, but not Th2 cells. **(A-C)** To investigate the degree of recruitment/infiltration of T cells, lumbar spinal cords were dissected from mice in each group on day 20–22 after immunization, analyzed by RT-PCR with primer for CD3 (A) and quantified (B), and analyzed by flow cytometry (C). Onset-treatment with HBPDS (30% ethanol extracted) significantly reduced the mRNA expression of CD3 (A and B) and the population of CD4^+^, CD4^+^/IFNγ^+^ (Th1) and CD4^+^/IL–17^+^ (Th17) cells, further increased the population of CD4^+^/CD25^+^/Foxp3^+^ and CD4^+^/Foxp3^+^/Helios^+^ (Treg) cells, and did not change the population CD4^+^/IL–4^+^ (Th2) cells in the lumbar spinal cords of EAE mice (C). Data are expressed as mean value ± SEM in the graph. (ANOVA test; #p < 0.05 and ##p < 0.01 compared to normal control mice, *p < 0.05 and **p < 0.01 compared to EAE mice).

Currently, the most important concept for the pathologic cascade of MS/EAE is that immunological self-tolerance is interrupted by autoreactive T cells. Peripheral autoreactive T cells traverse the blood brain barrier, activate the perivascular immune system, recruit and infiltrate into the CNS, and enlarge or repair the lesion in MS/EAE [25]. Therefore, the spinal cord was isolated from mice in each group after the peak day of neurological symptoms, and the population and activity of total T cells and helper T (Th) cells were examined by flow cytometry analysis and RT-PCR. First, the mRNA expression levels of CD3 (a marker of T cells) were significantly increased in the spinal cord of EAE mice (118.5 ± 4.9%) compared to normal control mice (49.0 ± 1.7%); however, the elevated mRNA expression was significantly reduced in the spinal cord of HBPDS-treated EAE mice (67.5 ± 10.1%) compared to EAE mice (Fig 9A and 9B). Furthermore, in the spinal cord of EAE mice, the percentage of CD4^+^ (Th) was markedly increased (Fig 9C; 6.91 ± 2.14%) compared to that in normal control mice (0.08 ± 0.06%). However, onset-treatment with HBPDS significantly reduced the increased population compared to those in EAE mice (Fig 9C; 6.91 ± 2.14% and 1.31 ± 0.36%, respectively). Products of Th1 and Th17 cells act as pro-inflammatory mediators and play a detrimental role in MS/EAE pathology, while products of Th2 and regulatory T (Treg) cells play a role in anti-inflammatory pathways including suppression of disease [1,2]. Therefore, we confirmed the effect of HBPDS on the population of CD4^+^ T cell subtype in the spinal cord of EAE mice (Fig 9C). The populations of CD4^+^/IFN-γ^+^ T (Th1) cells and CD4^+^/IL–17^+^ T (Th17) cells were markedly increased in the spinal cord of EAE mice compared to normal control mice (Fig 9C; 20.17 ± 3.76% and 13.40 ± 0.36%, respectively); however, the increased population was significantly reduced by onset-treatment with HBPDS compared to that in EAE mice (Fig 9C; 6.95 ± 2.23% and 4.35 ± 0.69%, respectively). Consecutively, the population of CD4-polarized Th2 and Treg cells was evaluated by flow cytometry in the spinal cord. The population of CD4^+^/IL–4^+^ (Th2) cells was not significantly changed in the spinal cord by immunization or onset-treatment with HBPDS (Fig 9C). Furthermore, we measured the percentage of Treg cells in the spinal cord. Interestingly, the population of CD4^+^/CD25^+^/Foxp3^+^ T cells was increased in the spinal cord of EAE mice (6.62 ± 0.52%) compared to normal control mice (3.33 ± 0.28%) or HBPDS alone-treated EAE mice (2.55 ± 0.41%). However, the population was further increased by onset-treatment with HBPDS (12.80 ± 3.34%) compared to that in EAE mice (Fig 9C). And then we discriminated whether the CD4^+^/CD25^+^/Foxp3^+^ Treg cells were derived from thymus using Helios (Ikaros family zinc-finger protein 2), a specific marker for thymus-derived naturally occurring Treg cells [26,27,28]. The population of CD4^+^/Foxp3^+^/Helios^+^ T cells was increased in the spinal cord of EAE mice (5.59 ± 0.72%) compared to normal control mice (3.27 ± 0.60%) or HBPDS alone-treated EAE mice (4.66 ± 0.21%), whereas, the increased population was further increased by HBPDS (9.06 ± 1.39%) compared to that in EAE mice (Fig 9C). The results suggest that HBPDS could reduce neurological signs of EAE by inhibiting the recruitment/infiltration of Th1 and Th17 cells into spinal cords of EAE mice and increasing the recruitment/infiltration of thymus-derived Treg cells into the spinal cords.

### 3.8. HBPDS stabilize the integrity of BBB in EAE mice

**Fig 10:**
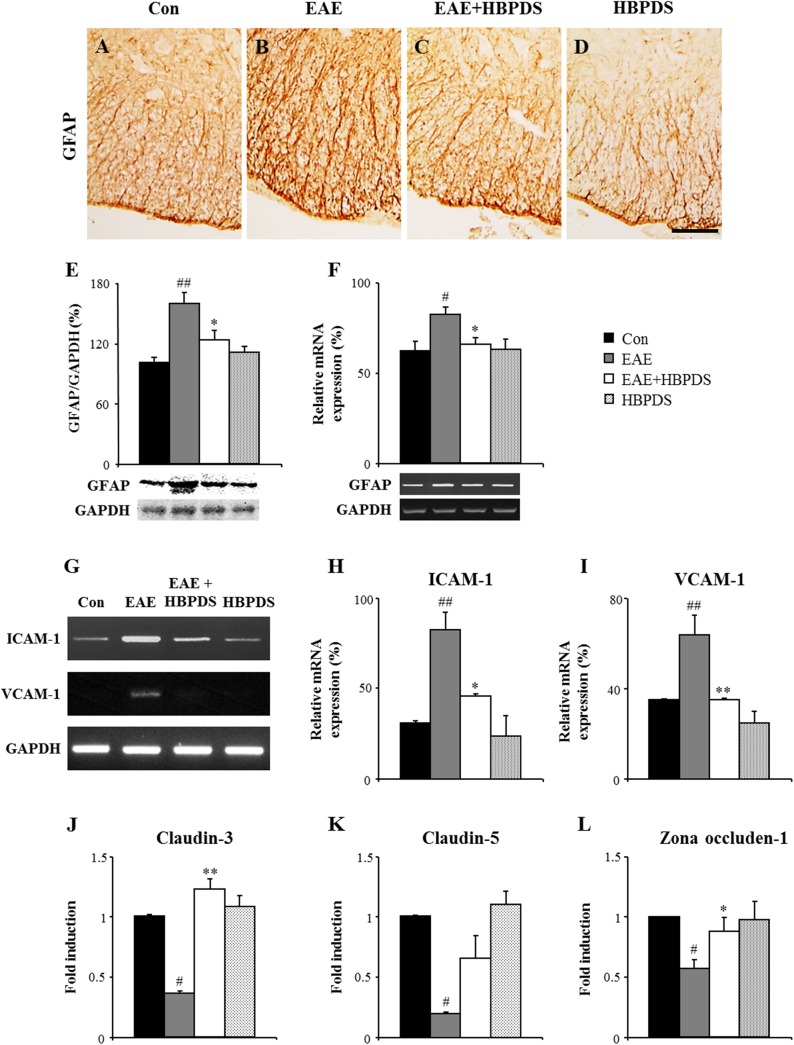
Onset-treatment with HBPDS helps to maintain the integrity of the BBB in EAE mice. **(A-L)** To assess the level of disruption of the BBB at day 20–22 after immunization, lumbar spinal cord sections was immunostained with anti-GFAP antibody (A-D), lumbar spinal cord lysate was analyzed by Western blot with anti-GFAP antibody (E), total RNA isolated from the lumbar spinal cord was analyzed by RT-PCR with primers for GFAP (F) and adhesive molecules (ICAM–1 and VCAM–1; G-I), and the total RNA was analyzed by real time-PCR with primers for junctional molecules (claudin–3, claudin–5, and zona occludens–1; J-L). Bands by Western blots and RT-PCR analysis were quantified as the ratio of GFAP to GAPDH for each sample (E and F). The mRNA expression level of each gene by real time-PCR is presented as the fold induction compared with the levels measured in normal mice (J-L). Onset-treatment with HBPDS (30% ethanol extracted) reduced the increased GFAP-immunoreactivity (A-D), the increased expression of protein and mRNA for GFAP (E and F), and the increased expression of mRNA for ICAM–1 and VCAM–1 (G-I), but increased the reduced expression of mRNA for claudin–3, claudin–5, and zona occludens–1 in the spinal cord of EAE mice (J-L). Data are represented as mean ± SEM. Scale bar = 100 μm. (ANOVA test; #p < 0.05 and ##p < 0.01 compared to normal control mice, *p < 0.05 and **p < 0.01 compared to EAE mice).

Neurological severity of EAE corresponds with the level of infiltration of inflammatory cells in the CNS by disruption of the BBB [3,29]. Since astrocytes are involved in formation of the BBB [30], we examined the effect of HBPDS on the level of astroglial activation in the spinal cord of EAE mice. GFAP immunoreactivity was increased in the white matter in the spinal cord of EAE mice compared to normal control mice (Fig 10A) or HBPDS alone-treated EAE mice (Fig 10D); however, the increased GFAP immunoreactivity was significantly reduced by onset-treatment with HBPDS compared to that in EAE mice (Fig 10C). These results corresponded with the changes in protein expression of GFAP by Western blot analysis (Fig 10E) and mRNA expression of GFAP by RT-PCR analysis (Fig 10F) in the spinal cord from EAE mice. To further investigate the effect of HBPDS on the integrity of the BBB, we measured the mRNA expression of adhesion (ICAM–1 and VCAM–1) and junctional molecules (claudin–3, claudin–5, and zona occludens–1) in the spinal cord of each group by RT-PCR (Fig 10G–10L). The mRNA expression of ICAM–1 and VCAM–1 was increased in the spinal cord of EAE mice compared to normal control mice; however, the increased expression was reduced by onset-treatment with HBPDS (Fig 10G–10I). The mRNA expression of claudin–3, claudin–5, and zona occludens–1 was decreased in the spinal cord of EAE mice compared to normal control mice; however, the decreased expression was up-regulated by onset-treatment with HBPDS (Fig 10J–10L). These results suggest that HBPDS inhibited the disruption of BBB in EAE mice and that this effect of HBPDS on stabilizing the BBB resulted in inhibition of migration and recruitment of peripheral immune cells (macrophages, T cells) across the endothelium.

### 3.9. Administration of HBPDS does not induce toxicity

Finally, to examine whether the treatment with HBPDS (extracted with 30% ethanol) induces toxicity in mice, we treated normal female mice (9-week-old) with 15, 30, and 60 mg/kg/day of HBPDS for 35 days and assessed the toxicity in blood, liver, spleen, and kidney (S1 Materials and Methods and S1 Fig). No evidence of a toxic effect was found, compared to that in normal mice without any saline. Mean body weight (S1A Fig) and mean serum level of the most commonly used indicators of liver damage or disease–AST, ALT, and LDH (S1B–S1D Fig) were not affected by prolonged administration of HBPDS. Also, the histological structure of the liver, spleen, and kidney was not affected by the administration of HBPDS (S1E–S1P Fig). The results indicated that prolonged administration of 15, 30, and 60 mg/kg/day of HBPDS does not induce toxicity.

## Discussion

In the present study, we demonstrated that HBPDS has a beneficial effect in EAE mice. Onset-treatment with HBPDS alleviates the severity of neurological signs and spinal demyelination, which were accompanied by inhibition of microglial activation, recruitment/infiltration of peripheral macrophages, expression of inflammatory mediators, and disruption of the BBB in the spinal cord of EAE mice. Onset-treatment with HBPDS inhibited the recruitment/infiltration of CD4^+^/ IFN-γ^+^ (Th1) and CD4^+^/IL–17^+^ (Th17) cells into spinal cords of EAE mice, while it increased the recruitment/infiltration of CD4^+^/CD25^+^/Foxp3^+^ and CD4^+^/CD25^+^/Helios^+^ T cells. Additionally, pre-, onset-, and post-treatment with HBPDS delayed or alleviated neurological signs in EAE mice. These findings suggest that HBPDS has a neuroprotective effect on the development and progression of EAE by regulating Th1, Th17, and (thymus-derived) Tregs responses.

In the past 20 years, significant progress has been made in the treatment of MS. Although more than 10 disease-modifying therapies (IFN-β, glatiramer acetate, mitoxantrone, natalizumab, fingolimod etc.) are now used for MS, they are not fully efficient and patients suffer from many symptoms because of the adverse effects and limitations of causal treatment [31,32]. Also, individuals with MS explore complementary and alternative medicine (CAM) therapies that are normally not prescribed by their conventional MS care providers, for improving the quality of life [33]. Therefore, research and development of new and safer treatment strategies based on CAM play a significant role in the well being of MS patients. Biologically based therapies include herbs, diet, and bee venom therapies (Kim et al., 2009; Landis et al., 2012). *Hypericum Perforatum*, *Valeriana Officinalis*, *Ginkgo biloba*, *Panax ginseng*, *and Curcuma longa* are commonly used in the treatment of MS [33]. In the present study, HBPDS, which is an herbal medicine for treating influenza and for enhancing immune activity in Oriental medicine, displayed a beneficial effect in MOG-induced EAE. Although its efficacy when used for onset-treatment differed slightly based on the type of solvent (water, 30%, 70%, and absolute ethanol), it was approximately similar and the representative HBPDS extract (30% ethanol) had a beneficial effect when used for pre-, onset-, and post-treatment. These results indicate that HBPDS may provide the basis for new and safer neuroprotective strategies for MS, and a further study including the investigation of active single herbs and active ingredients is needed.

CD4^+^ T cells producing IL–17 are related to inflammatory reactions in many autoimmune diseases, which have been defined as Th17 cells. It has been revealed initially that murine Th17 cells are differentiated by a combination of TGF-β and IL–6 cytokines, whereas IL–23 is executed for their recruitment and amplification [34]. Recent studies have demonstrated that TGF-β elevates Th17 cells by suppressing the differentiation of Th1 and Th2 cells. Furthermore, TGF-β has been considered an important factor in the immunosuppression of effecter T cells such as Th1 and in the induction of immune tolerance, and an immune-regulation cytokine produced by some Foxp3- and/or Helios-expressing Treg cells [27,28,34,35,36]. Helios is selectively induced in FoxP3^+^ Treg cells and regulate Th cell differentiation and cytokine production [26,27,28]. Moreover, Helios is a specific marker for thymus-derived naturally occurring Treg cells but not for peripherally induced Treg cells [26,27,28]. Treg cells play a crucial role in the maintenance of peripheral immune tolerance and can regulate effecter T cell-mediated autoimmune inflammation directly or via the antigen-presenting cells during MS/EAE [37,38,39]. In the present study, the increased population of CD4^+^/IFNγ^+^ T (Th1) cells and CD4^+^/IL–17^+^ T (Th17) cells in the spinal cord from EAE mice was significantly diminished by onset-treatment with HBPDS, while the increased population of CD4^+^/CD25^+^/Foxp3^+^ and CD4^+^/CD25^+^/Helios^+^ T (Treg) cells in the spinal cord from EAE mice was further increased by onset-treatment with HBPDS. However, the population of CD4^+^/IL–4^+^ T (Th2) cells in the spinal cords of EAE mice was not significantly changed by immunization or onset-treatment with HBPDS. These results indicate that onset-treatment with HBPDS suppresses the recruitment/infiltration of Th1 and Th17 cells and increases the recruitment/migration of thymus-derived Treg cells into the spinal cord of EAE mice.

Under steady-state conditions, resident microglia are not influenced by bone marrow-derived macrophages. However, on CNS inflammation, macrophages migrate and infiltrate into the lesion of the CNS, have a similar phenotype to activated microglia, and express the same antigenic markers [24,40]. Therefore, we assessed the distribution of macrophages and microglia in the spinal cord of EAE mice using flow cytometry. The number of macrophages (CD11b^+^/CD45^+/high^) and microglia (CD11b^+^/CD45^+/low^) was markedly increased to 12.39 ± 1.21% and 3.35 ± 0.86%, respectively in the spinal cord of EAE mice; however, these increases were significantly attenuated to 2.12 ± 1.27% and 1.73 ± 0.64%, respectively by onset-treatment with HBPDS (Fig 8G and 8H). The results indicate that onset-treatment with HBPDS suppresses the recruitment and infiltration of macrophages/microglia in the spinal cord of EAE mice, corresponding with the results of the histological analysis (H&E stain and immunohistochemistry for Iba–1). The infiltrated immune cells contribute to the process of tissue damage and repair by producing pro- and anti-inflammatory mediators [22,24,40,41]. In the present study, onset-treatment with HBPDS significantly increased the reduced expression levels of representative cytokines (TNF-α, IL–1β, IL–6), representative chemokines (MCP–1, MIP–1α, and RANTES), and iNOS in the spinal cord from EAE mice (Fig 7), corresponding with the histopathological results (demyelination and infiltration of immune cells). These findings support a beneficial effect of HBPDS on spinal demyelination in EAE mice. Also, our findings indicate that reduction in Iba–1 immunoreactivity in the spinal cord from HBPDS-treated EAE mice (Fig 8) is associated with reduction in activation and infiltration of residential microglia as well as macrophages. Taken together, it is likely that onset-treatment with HBPDS may contribute to preventing the development and progression of EAE by inhibiting recruitment/infiltration of immune cells into demyelinating lesions and secretion of pro-inflammatory mediators such as cytokines and chemokines in the spinal cord.

In neurological diseases such as MS and stroke, activated astrocytes and leukocytes secrete pro-inflammatory modulators, resulting in loss of the BBB integrity and increase in cell adhesion molecules (CAMs). During MS and EAE, the increased expression of CAMs (ICAM–1 and VCAM–1) plays an important role in leukocyte transmigration into the CNS [2]. In this study, onset-treatment with HBPDS reduced the increased immunoreactivity, protein expression, and mRNA expression of GFAP and the increased mRNA expression of ICAM–1 and VCAM–1 in the spinal cord of EAE mice, corresponding to the reduced clinical signs of EAE and infiltration of immune cells in the spinal cord. For maintaining the integrity of the BBB, tight junction-associated molecules (claudin–3, claudin–5, and zona occludens–1) combine among the endothelial cells. In EAE, the expression of junctional molecules is suppressed, which results in increased BBB permeability [42]. In the present study, onset-treatment with HBPDS increased the reduced mRNA expression of claudin–3, claudin–5, and zona occludens–1 in the spinal cord of EAE mice. The results suggest that HBPDS helps to preserve the integrity of the BBB in EAE mice.

All herbs that constitute HBPDS are well-known herbs in East Asian traditional medicine, and have some potent biological activities. *Ostericum koreanum* has been traditionally prescribed as an analgesic and cold medication. Isolated oxypeucedanin has anti-cancer effects through the regulation of cell cycle arrest and apoptosis [43]. And bisabolangelone, also isolated from the roots of *Ostericum koreanum*, has anti-inflammatory effects on lipopolysaccharide-stimulated inflammation by inhibiting the NF-κB and MAPKs pathways in macrophages [44]. *Aralia continentalis* is an ethnomedicinal plant traditionally prescribed to treat headaches, rheumatism, lumbago, and limping [45]. Its extract has a protective effect against carbon tetrachloride-induced hepatotoxicity by promoting anti-oxidative protein expression [46] and anti-nociceptive effects in complete Freund’s adjuvant-induced arthritis [47]. Moreover, kaurenoic acid isolated from *Aralia continentalis* suppresses the lipopolysaccharide-induced inflammatory response in RAW264.7 macrophages through the inhibition of iNOS and COX–2 expression [48]. *Bupleurum falcatum* has been used in Chinese medicine as a liver tonic [49]. Its extract has anti-anxiety activity after repeated restraint stress [50], antidepressant-like effect in the tail suspension test [51], protective effect against allergic asthma [52], and neuroprotective and anti-inflammatory effects after spinal cord injury by inhibiting the activation of MMPs [53]. *Angelica decursiva* or isolated umbelliferone 6-carboxylic acid has anti-oxidant and anti-inflammatory activities *in vitro* using lipopolysaccharide-stimulated RAW264.7 cells [54,55]. *Schizonepeta tenuifolia* has a wide range of the physiological activities against inflammation-related diseases and oxidative stress [56,57,58]. *Saposhnikovia divaricata* is used to treat rheumatoid arthritis via inhibiting NF-κB and MARKs [59], and has potent antioxidant, anti-inflammatory, and protective properties on lipopolysaccharide-activated RAW 264.7 cells [60]. *Poria cocos* has been traditionally used in Oriental medicine as a diuretic and sedative [61] and 25-methoxyporicoic acid A and other triterpene acids from *Poria cocos* have anti-tumor-promoting effects [62]. *Rehmannia glutinosa* belongs to the family of Scrophulariaceae, and has been widely used to treat enervation in many Asian countries. It promotes the proliferation of hematopoietic stem cells in bone marrow [63]. Furthermore, a compound formula that includes *Rehmannia glutinosa* alleviates levodopa-induced dyskinesia in Parkinson’s disease [64]. *Lycium barbarum* protects against neuronal loss, excitatory cytotoxicity induced by glutamate, and ameliorates cognitive and memory deficits induced by scopolamine [65]. *Plantago asiatica* has therapeutic effects including anti-fever, anti-viral, anti-oxidant, and anti-cancer activities [66,67]. Although it remains still unknown which herb provides the advantageous activity for MS/EAE, our present results are an important first step towards a better understanding.

## Conclusions

Although HBPDS has been traditionally prescribed for various physiological and immunological disorders, its effects on the pathogenesis and progression of MS/EAE have not been established. In this study, onset-treatment with HBPDS suppressed neurological symptoms, demyelination, infiltration and activation of microglia and macrophages, and expression of pro-inflammatory cytokines and chemokines in the spinal cords of EAE mice. Onset-treatment with HBPDS reduced the loss of adhesive and junctional molecules to maintain BBB integrity of EAE mice. Onset-treatment with HBPDS down-regulated the populations of CD4^+^/IFN-γ^+^ and CD4^+^/IL–17^+^ T cells, and up-regulated the population of CD4^+^/CD25^+^/Foxp3^+^ and CD4^+^/Foxp3^+^/Helios^+^ Treg cells in the both the spinal cord of EAE mice. These results suggest that HBPDS exerted the novel neuroprotective and anti-inflammatory properties on MOG-induced EAE by inhibiting the myelin-reactive T cells and immune modulators. We are carefully optimistic that HBPDS could be used as a targeted neuroprotective strategy for autoimmune diseases such as MS.

## Supporting Information

S1 Fig (TIF)

S1 Table (TIF)

S1 Materials and Methods (DOC)

## References

[1] Lassmann H, van Horssen J (2011) The molecular basis of neurodegeneration in multiple sclerosis. FEBS Lett 585: 3715–3723. 10.1016/j.febslet.2011.08.004 21854776

[2] Alvarez JI, Cayrol R, Prat A (2011) Disruption of central nervous system barriers in multiple sclerosis. Biochim Biophys Acta 1812: 252–264. 10.1016/j.bbadis.2010.06.017 20619340

[3] Kawakami N, Bartholomaus I, Pesic M, Mues M (2012) An autoimmunity odyssey: how autoreactive T cells infiltrate into the CNS. Immunol Rev 248: 140–155. 10.1111/j.1600-065X.2012.01133.x 22725959

[4] Finkelsztejn A (2014) Multiple sclerosis: overview of disease-modifying agents. Perspect Medicin Chem 6: 65–72. 10.4137/PMC.S13213 25336899 PMC4197902

[5] Minagar A (2013) Current and future therapies for multiple sclerosis. Scientifica (Cairo) 2013: 249101. 24278770 10.1155/2013/249101 PMC3820353

[6] Liu JP, Feng L, Zhang MH, Ma DY, Wang SY, Gu J, et al (2013) Neuroprotective effect of Liuwei Dihuang decoction on cognition deficits of diabetic encephalopathy in streptozotocin-induced diabetic rat. J Ethnopharmacol 150: 371–381. 10.1016/j.jep.2013.09.003 24041458

[7] Song LJ, Y.P. F (2010) Influence of treatment based on syndrome differentiation mainly with tonifying the kidney on cytokines in plasm of multiple sclerosis at acute stage China Journal of Traditional Chinese Medicine and Pharmacy 25: 745–748

[8] Fan YP, Wang P, Zhang XH, Gong HY, Zhou L, Liu XZ, et al (2006) Treatment of relapsing multiple sclerosis with Erhuang Formula Journal of Beijing University on Traditional Chinese Medicine 29: 273–276.

[9] Kou S, Zheng Q, Wang Y, Zhao H, Zhang Q, LI M, et al (2014) Zuo-Gui and You-Gui pills, two traditional Chinese herbal formulas, downregulated the expression of NogoA, NgR, and RhoA in rats with experimental autoimmune encephalomyelitis. J Ethnopharmacol 158 Pt A: 102–112. 10.1016/j.jep.2014.10.007 25448504

[10] Wang L, Fan YP, Gong HY, Ye M, Zhou L, Zhao H, et al (2008) Effect of Zuogui Pill and Yougui Pill on remyelination and axonal regeneration in rats with experimental allergic encephalomyelitis China Journal of Experimental Traditional Medical Formulae 4: 42–45.

[11] Park JW, Lee BW, Bae JU (2012) Extended indications of Soyang-type Hyeongbangpaedok-san using Dongeuibogam. Journal of Oriental Medical Classics 25: 17–29.

[12] Lee SY, Ahn TW (2005) Effects of Hyeongbangpaedok-san and Dokhwaljihwang-tang that get weight, hematology, biochemistry change by wistar rat’s aging. J Sasang Constitut Med 17: 91–102.

[13] Heo JW, Kang H, Ahn KS, Kim SH, Choi SH, Ahn KS, et al (2009) Study on the Anti-inflammatory Effects of Soyangin-Hyeongbangpaedok-san. Korean journal of oriental physiology & pathology 23: 443–451.

[14] Kim JB, Kang H, Ahn KS, Shim BS, Kim SH, Choi SH, et al (2009) Effect of Soyangin-Hyeongbangpaedok-san on Anti-CD3 Stimulated Mouse T Cells In Vivo and In Vitro. Korean journal of oriental physiology & pathology 23.

[15] Landis SC, Amara SG, Asadullah K, Austin CP, Blumenstein R, Bradley EW, et al (2012) A call for transparent reporting to optimize the predictive value of preclinical research. Nature 490: 187–191. 10.1038/nature11556 23060188 PMC3511845

[16] Lee MJ, Jang M, Choi J, Lee G, Min HJ, Chung WS, et al (2015) Bee Venom Acupuncture Alleviates Experimental Autoimmune Encephalomyelitis by Upregulating Regulatory T Cells and Suppressing Th1 and Th17 Responses. Mol Neurobiol. 10.1007/s12035-014-9012-2 25579380

[17] Lee MJ, Jang M, Choi J, Chang BS, Kim DY, Kim SH, et al (2015) Korean red ginseng and ginsenoside-Rb1/-Rg1 alleviates experimental autoimmune encephalomyelitis by suppressing Th1 and Th17 T cells and upregulating regulatory T cells. Molecular Neurobiology. 10.1007/s12035-015-9131-4 25846819

[18] Fissolo N, Costa C, Nurtdinov RN, Bustamante MF, Llombart V, Mansilla MJ, et al (2012) Treatment with MOG-DNA vaccines induces CD4+CD25+FoxP3+ regulatory T cells and up-regulates genes with neuroprotective functions in experimental autoimmune encephalomyelitis. J Neuroinflammation 9: 139 10.1186/1742-2094-9-139 22727044 PMC3464883

[19] Jang M, Lee MJ, Cho IH (2014) Ethyl pyruvate ameliorates 3-nitropropionic acid-induced striatal toxicity through anti-neuronal cell death and anti-inflammatory mechanisms. Brain Behav Immun 38: 151–165. 10.1016/j.bbi.2014.01.015 24576481

[20] Jahromi SR, Arrefhosseini SR, Ghaemi A, Alizadeh A, Sabetghadam F, Togha M (2014) Effect of oral genistein administration in early and late phases of allergic encephalomyelitis. Iran J Basic Med Sci 17: 509–515. 25429342 PMC4242921

[21] Jahromi SR, Arrefhosseini SR, Ghaemi A, Alizadeh A, Sabetghadam F, Togha M (2011) Effect of oral genistein administration in early and late phases of allergic encephalomyelitis. Iran J Basic Med Sci 17: 509–515. PMC4242921 25429342

[22] Gao Z, Tsirka SE (2011) Animal Models of MS Reveal Multiple Roles of Microglia in Disease Pathogenesis. Neurol Res Int 2011: 383087 10.1155/2011/383087 22203900 PMC3238412

[23] Jonas RA, Yuan TF, Liang YX, Jonas JB, Tay DK, Ellis-Behnke RG (2012) The spider effect: morphological and orienting classification of microglia in response to stimuli in vivo. PLoS One 7: e30763 10.1371/journal.pone.0030763 22363486 PMC3283598

[24] Rawji KS, Yong VW (2013) The benefits and detriments of macrophages/microglia in models of multiple sclerosis. Clin Dev Immunol 2013: 948976 10.1155/2013/948976 23840244 PMC3694375

[25] Petermann F, Korn T (2011) Cytokines and effector T cell subsets causing autoimmune CNS disease. FEBS Lett 585: 3747–3757. 10.1016/j.febslet.2011.03.064 21477588

[26] Thornton AM, Korty PE, Tran DQ, Wohlfert EA, Murray PE, Belkaid Y, et al (2010) Expression of Helios, an Ikaros transcription factor family member, differentiates thymic-derived from peripherally induced Foxp3+ T regulatory cells. J Immunol 184: 3433–3441. 10.4049/jimmunol.0904028 20181882 PMC3725574

[27] Lin X, Chen M, Liu Y, Guo Z, He X, Brand D, et al (2013) Advances in distinguishing natural from induced Foxp3(+) regulatory T cells. Int J Clin Exp Pathol 6: 116–123. 23329997 PMC3544233

[28] Takatori H, Kawashima H, Matsuki A, Meguro K, Tanaka S, Iwamoto T, et al (2015) Helios Enhances Treg Cell Function in Cooperation With FoxP3. Arthritis Rheumatol 67: 1491–1502. 10.1002/art.39091 25733061

[29] Fabis MJ, Scott GS, Kean RB, Koprowski H, Hooper DC (2007) Loss of blood-brain barrier integrity in the spinal cord is common to experimental allergic encephalomyelitis in knockout mouse models. Proc Natl Acad Sci U S A 104: 5656–5661. 17372191 10.1073/pnas.0701252104 PMC1838442

[30] Willis CL (2010) Glia-induced reversible disruption of blood-brain barrier integrity and neuropathological response of the neurovascular unit. Toxicol Pathol 39: 172–185. 10.1177/0192623310385830 21189317

[31] Chen SJ, Wang YL, Fan HC, Lo WT, Wang CC, Sytwu HK (2012) Current status of the immunomodulation and immunomediated therapeutic strategies for multiple sclerosis. Clin Dev Immunol 2012: 970789 10.1155/2012/970789 22203863 PMC3235500

[32] Cuzzola VF, Palella E, Celi D, Barresi M, Giacoppo S, Marino S (2012) Pharmacogenomic update on multiple sclerosis: a focus on actual and new therapeutic strategies. Pharmacogenomics J 12: 453–461. 10.1038/tpj.2012.41 23044601

[33] Namjooyan F, Ghanavati R, Majdinasab N, Jokari S, Janbozorgi M (2014) Uses of complementary and alternative medicine in multiple sclerosis. J Tradit Complement Med 4: 145–152. 10.4103/2225-4110.136543 25161918 PMC4142451

[34] Mangan PR, Harrington LE, O'Quinn DB, Helms WS, Bullard DC, Elson CO, et al (2006) Transforming growth factor-beta induces development of the T(H)17 lineage. Nature 441: 231–234. 16648837 10.1038/nature04754

[35] Chen W, Jin W, Hardegen N, Lei KJ, Li L, Marinos N, et al (2003) Conversion of peripheral CD4+CD25- naive T cells to CD4+CD25+ regulatory T cells by TGF-beta induction of transcription factor Foxp3. J Exp Med 198: 1875–1886. 14676299 10.1084/jem.20030152 PMC2194145

[36] Wan YY, Flavell RA (2005) Identifying Foxp3-expressing suppressor T cells with a bicistronic reporter. Proc Natl Acad Sci U S A 102: 5126–5131. 15795373 10.1073/pnas.0501701102 PMC556008

[37] Sakaguchi S (2000) Regulatory T cells: key controllers of immunologic self-tolerance. Cell 101: 455–458. 10850488 10.1016/s0092-8674(00)80856-9

[38] Liston A, Rudensky AY (2007) Thymic development and peripheral homeostasis of regulatory T cells. Curr Opin Immunol 19: 176–185. 17306520 10.1016/j.coi.2007.02.005

[39] Fletcher JM, Lalor SJ, Sweeney CM, Tubridy N, Mills KH (2010) T cells in multiple sclerosis and experimental autoimmune encephalomyelitis. Clin Exp Immunol 162: 1–11. 10.1111/j.1365-2249.2010.04143.x 20682002 PMC2990924

[40] Ajami B, Bennett JL, Krieger C, McNagny KM, Rossi FM (2011) Infiltrating monocytes trigger EAE progression, but do not contribute to the resident microglia pool. Nat Neurosci 14: 1142–1149. 10.1038/nn.2887 21804537

[41] London A, Cohen M, Schwartz M (2013) Microglia and monocyte-derived macrophages: functionally distinct populations that act in concert in CNS plasticity and repair. Front Cell Neurosci 7: 34 10.3389/fncel.2013.00034 23596391 PMC3625831

[42] Lanz TV, Becker S, Osswald M, Bittner S, Schuhmann MK, Opitz CA, et al (2013) Protein kinase Cbeta as a therapeutic target stabilizing blood-brain barrier disruption in experimental autoimmune encephalomyelitis. Proc Natl Acad Sci U S A 110: 14735–14740. 10.1073/pnas.1302569110 23959874 PMC3767524

[43] Kang TJ, Lee SY, Singh RP, Agarwal R, Yim DS (2009) Anti-tumor activity of oxypeucedanin from Ostericum koreanum against human prostate carcinoma DU145 cells. Acta Oncol 48: 895–900. 10.1080/02841860902824925 19322700

[44] Jung HW, Mahesh R, Park JH, Boo YC, Park KM, Park YK (2010) Bisabolangelone isolated from Ostericum koreanum inhibits the production of inflammatory mediators by down-regulation of NF-kappaB and ERK MAP kinase activity in LPS-stimulated RAW264.7 cells. Int Immunopharmacol 10: 155–162. 10.1016/j.intimp.2009.10.010 19879381

[45] LI SZ (1578) Ben Cao Gang Mu (《本草纲目》.

[46] Hwang YP, Choi JH, Jeong HG (2009) Protective effect of the Aralia continentalis root extract against carbon tetrachloride-induced hepatotoxicity in mice. Food Chem Toxicol 47: 75–81. 10.1016/j.fct.2008.10.011 18984026

[47] Park HJ, Hong MS, Lee JS, Leem KH, Kim CJ, Kim JW, et al (2005) Effects of Aralia continentalis on hyperalgesia with peripheral inflammation. Phytother Res 19: 511–513. 16114085 10.1002/ptr.1693

[48] Choi RJ, Shin EM, Jung HA, Choi JS, Kim YS (2011) Inhibitory effects of kaurenoic acid from Aralia continentalis on LPS-induced inflammatory response in RAW264.7 macrophages. Phytomedicine 18: 677–682. 10.1016/j.phymed.2010.11.010 21211951

[49] Huh J (1610) Donguibogam. Seoul: Namsandang Press.

[50] Lee B, Yun HY, Shim I, Lee H, Hahm DH (2012) Bupleurum falcatum prevents depression and anxiety-like behaviors in rats exposed to repeated restraint stress. J Microbiol Biotechnol 22: 422–430. 22450800 10.4014/jmb.1110.10077

[51] Kwon S, Lee B, Kim M, Lee H, Park HJ, Hahm DH (2010) Antidepressant-like effect of the methanolic extract from Bupleurum falcatum in the tail suspension test. Prog Neuropsychopharmacol Biol Psychiatry 34: 265–270. 10.1016/j.pnpbp.2009.11.015 19932727

[52] Park KH, Park J, Koh D, Lim Y (2002) Effect of saikosaponin-A, a triterpenoid glycoside, isolated from Bupleurum falcatum on experimental allergic asthma. Phytother Res 16: 359–363. 12112293 10.1002/ptr.903

[53] Lee JY, Kim HS, Oh TH, Yune TY (2010) Ethanol Extract of Bupleurum falcatum Improves Functional Recovery by Inhibiting Matrix Metalloproteinases–2 and -9 Activation and Inflammation after Spinal Cord Injury. Exp Neurobiol 19: 146–154. 10.5607/en.2010.19.3.146 22110354 PMC3214781

[54] Zhao D, Islam MN, Ahn BR, Jung HA, Kim BW, Choi JS (2012) In vitro antioxidant and anti-inflammatory activities of Angelica decursiva. Arch Pharm Res 35: 179–192. 10.1007/s12272-012-0120-0 22297757

[55] Islam MN, Choi RJ, Jin SE, Kim YS, Ahn BR, Zhao D, et al (2012) Mechanism of anti-inflammatory activity of umbelliferone 6-carboxylic acid isolated from Angelica decursiva. J Ethnopharmacol 144: 175–181. 10.1016/j.jep.2012.08.048 22981803

[56] Kim SJ, Kim JS, Choi IY, Kim DH, Kim MC, An HJ, et al (2008) Anti-inflammatory activity of Schizonepeta tenuifolia through the inhibition of MAPK phosphorylation in mouse peritoneal macrophages. Am J Chin Med 36: 1145–1158. 19051342 10.1142/S0192415X0800648X

[57] Byun MW (2013) Schizonepeta tenuifolia ethanol extract exerts anti-inflammatory activity through the inhibition of TLR4 signaling in lipopolysaccharide-stimulated macrophage cells. J Med Food 17: 350–356. 10.1089/jmf.2013.2928 24650252

[58] Wang BS, Huang GJ, Tai HM, Huang MH (2012) Antioxidant and anti-inflammatory activities of aqueous extracts of Schizonepeta tenuifolia Briq. Food Chem Toxicol 50: 526–531. 10.1016/j.fct.2011.12.010 22198607

[59] Kong X, Liu C, Zhang C, Zhao J, Wang J, Wan H, et al (2013) The suppressive effects of Saposhnikovia divaricata (Fangfeng) chromone extract on rheumatoid arthritis via inhibition of nuclear factor-kappaB and mitogen activated proteinkinases activation on collagen-induced arthritis model. J Ethnopharmacol 148: 842–850. 10.1016/j.jep.2013.05.023 23711830

[60] Tai J, Cheung S (2007) Anti-proliferative and antioxidant activities of Saposhnikovia divaricata. Oncol Rep 18: 227–234. 17549372

[61] Zhao YY, Feng YL, Du X, Xi ZH, Cheng XL, Wei F (2012) Diuretic activity of the ethanol and aqueous extracts of the surface layer of Poria cocos in rat. J Ethnopharmacol 144: 775–778. 10.1016/j.jep.2012.09.033 23058989

[62] Akihisa T, Uchiyama E, Kikuchi T, Tokuda H, Suzuki T, Kimura Y (2009) Anti-tumor-promoting effects of 25-methoxyporicoic acid A and other triterpene acids from Poria cocos. J Nat Prod 72: 1786–1792. 10.1021/np9003239 19746919

[63] Wang YB, Liu YF, Lu XT, Yan FF, Wang B, Bai WW, et al (2013) Rehmannia glutinosa extract activates endothelial progenitor cells in a rat model of myocardial infarction through a SDF–1 alpha/CXCR4 cascade. PLoS One 8: e54303 10.1371/journal.pone.0054303 23349848 PMC3548813

[64] Teng L, Hong F, Zhang C, He J, Wang H (2014) Compound Formula Rehmannia alleviates levodopa-induced dyskinesia in Parkinson's disease. Neural Regen Res 9: 407–412. 10.4103/1673-5374.128246 25206828 PMC4146199

[65] Chen W, Cheng X, Chen J, Yi X, Nie D, Sun X, et al (2014) Lycium barbarum polysaccharides prevent memory and neurogenesis impairments in scopolamine-treated rats. PLoS One 9: e88076 10.1371/journal.pone.0088076 24505383 PMC3914900

[66] Chiang LC, Chiang W, Chang MY, Lin CC (2003) In vitro cytotoxic, antiviral and immunomodulatory effects of Plantago major and Plantago asiatica. Am J Chin Med 31: 225–234. 12856861 10.1142/S0192415X03000874

[67] Ye CL, Hu WL, Dai DH (2011) Extraction of polysaccharides and the antioxidant activity from the seeds of Plantago asiatica L. Int J Biol Macromol 49: 466–470. 10.1016/j.ijbiomac.2011.05.026 21664928

