# Correction: An Oriental Medicine, Hyungbangpaedok-San Attenuates Motor Paralysis in an Experimental Model of Multiple Sclerosis by Regulating the T Cell Response

**DOI:** 10.1371/journal.pone.0141635

**Published:** 2015-10-22

**Authors:** The PLOS ONE Staff

There are errors in the Funding section. The publisher apologizes for these errors. The correct funding information is as follows: Original study was supported by the Traditional Korean Medicine R&D program founded by the Ministry of Health & Welfare through the Korea Health Industry Development Institute (KHIDI) (HI13C0263) and Basic Science Research Program through the National Research Foundation of Korea (NRF) funded by the Ministry of Education, Science and Technology (NRF-2014R1A2A1A11051240). And study for revision was supported by “Cooperative Research Program for Agriculture Science & Technology Development (Project No. PJ011582042015)” Rural Development Administration, Republic of Korea. The funders had no role in study design, data collection and analysis, decision to publish, or preparation of the manuscript.
