# Supplementary figures and images for: An Oriental Medicine, Hyungbangpaedok-San Attenuates Motor Paralysis in an Experimental Model of Multiple Sclerosis by Regulating the T Cell Response
Source: PLoS One. 2015 Oct 7;10(10):e0138592. doi: 10.1371/journal.pone.0138592 (PMC4596626; doi:10.1371/journal.pone.0138592)

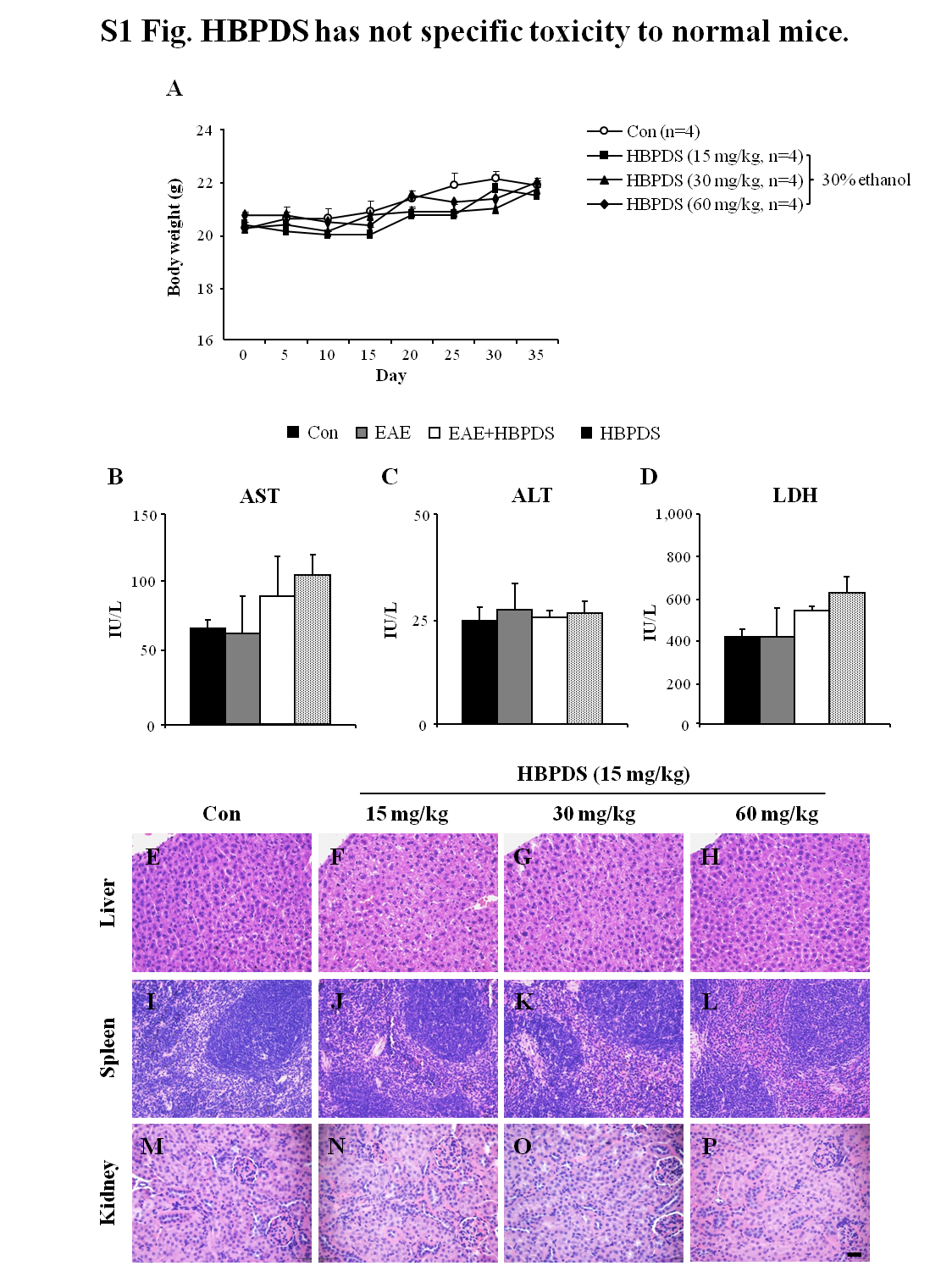

Supplement: S1 Fig — (TIF) [file pone.0138592.s001.tif]

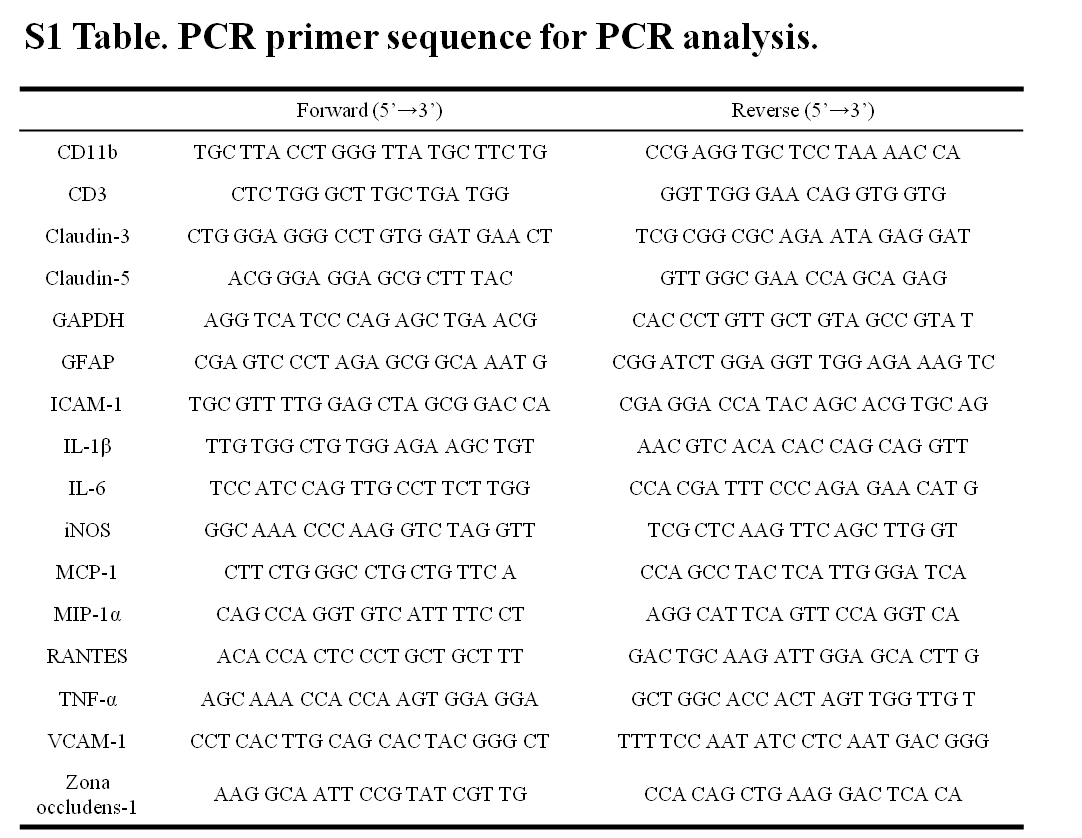

Supplement: S1 Table — (TIF) [file pone.0138592.s002.tif]
