# Supplementary material for: An Oriental Medicine, Hyungbangpaedok-San Attenuates Motor Paralysis in an Experimental Model of Multiple Sclerosis by Regulating the T Cell Response
Source: PLoS One. 2015 Oct 7;10(10):e0138592. doi: 10.1371/journal.pone.0138592 (PMC4596626; doi:10.1371/journal.pone.0138592)
Supplement: S1 Materials and Methods — (DOC) [file pone.0138592.s003.doc]

**Supporting Information**

**S1 Materials and Methods. Toxicological evaluation of HBPDS.** To examine whether HBPDS has a toxic effect for long-term use in mice, 30% ethanol-extracted HBPDS (15, 30, and 60 mg/kg/day) was orally administrated to normal female mice (9-week-old) for 35 days. Body weight was measured and serum was obtained at 24 hours after the last administration of HBPDS. Serum level of alanine aminotransferase (ALT), aspartate aminotransferase (AST), and lactate dehydrogenase (LDH) was measured using an enzymatic or ultraviolet assay with ALT, AST, or LDH detection kit (Roche, Basel, Switzerland) according to the manufacturer's instructions under cobas 8000 modular analyzer (Roche, Basel, Swetzerland) and the previously described protocol [19]. General histological structure was evaluated by H&E staining according to pre-described protocols.

**S1 Fig. HBPDS has not specific toxicity to normal mice.** **(A-P)** To examine whether the treatment of HBPDS induces toxicity to mice, normal female, 9-week-old mice were treated with 15, 30, and 60 mg/kg/day of HBPDS (30% ethanol extracted) for 35 days and toxicity was assessed in blood, liver, spleen, and kidney. No evidence of a toxic effect was evident, compared to those of normal mice without any saline. Mean body weight (A) and mean serum level of AST (B), ALT (C), and LDH (D) were not affected by the treatment of HBPDS for long-term. Also, histological structure liver (E-H), spleen (I-L), and kidney (M-P) were not significantly affected by the treatment of HBPDS. Data are represented as mean ± SEM.

**S1 Table. PCR primer sequence for PCR analysis.**
